# Enhanced Probiotic Potential of *Lactobacillus reuteri* When Delivered as a Biofilm on Dextranomer Microspheres That Contain Beneficial Cargo

**DOI:** 10.3389/fmicb.2017.00489

**Published:** 2017-03-27

**Authors:** Jason B. Navarro, Lauren Mashburn-Warren, Lauren O. Bakaletz, Michael T. Bailey, Steven D. Goodman

**Affiliations:** ^1^Center for Microbial Pathogenesis, The Research Institute at Nationwide Children's HospitalColumbus, OH, USA; ^2^Wexner Medical Center, Institute for Behavioral Medicine Research, The Ohio State UniversityColumbus, OH, USA

**Keywords:** *Lactobacillus reuteri*, microsphere, reuterin, glucosyltransferase, maltose, dextranomer

## Abstract

As with all orally consumed probiotics, the Gram-positive bacterium *Lactobacillus reuteri* encounters numerous challenges as it transits through the gastrointestinal tract of the host, including low pH, effectors of the host immune system, as well as competition with commensal and pathogenic bacteria, all of which can greatly reduce the availability of live bacteria for therapeutic purposes. Recently we showed that *L. reuteri*, when adhered in the form of a biofilm to a semi-permeable biocompatible dextranomer microsphere, reduces the incidence of necrotizing enterocolitis by 50% in a well-defined animal model following delivery of a single prophylactic dose. Herein, using the same semi-permeable microspheres, we showed that providing compounds beneficial to *L. reuteri* as diffusible cargo within the microsphere lumen resulted in further advantageous effects including glucosyltransferase-dependent bacterial adherence to the microsphere surface, resistance of bound bacteria against acidic conditions, enhanced adherence of *L. reuteri* to human intestinal epithelial cells *in vitro*, and facilitated production of the antimicrobial compound reuterin and the anti-inflammatory molecule histamine. These data support continued development of this novel probiotic formulation as an adaptable and effective means for targeted delivery of cargo beneficial to the probiotic bacterium.

## Introduction

Probiotic bacteria are “live microorganisms that, when administered in adequate amounts, confer a health benefit on the host” (Araya et al., 2006). Commercially, probiotics can be diverse genera of bacteria both alone and in combination are utilized for the treatment of numerous ailments and diseases, such as diarrheal diseases (McFarland, 2006; Johnston et al., 2012), infant colic (Savino et al., 2010; Sung et al., 2014), allergies (Soh et al., 2009; Stefka et al., 2014), and elevated LDL-cholesterol (Agerholm-Larsen et al., 2000; Nguyen et al., 2007).

Orally consumed beneficial bacteria face a gauntlet of challenges in the host, such as low pH in the stomach, effectors of the host immune system and competition with commensal and pathogenic bacteria (Ding and Shah, 2007). All of these factors negatively impact the ability of ingested probiotic bacteria to be sufficiently sustained within the host and thus reduce the potentially beneficial effects conferred. Commercially, encapsulation of lyophilized probiotics is used as the primary means to protect bacterial viability (Cook et al., 2012; Kailasapathy, 2014), however there is no evidence of improved probiotic persistence within the host.

**Figure 1:**
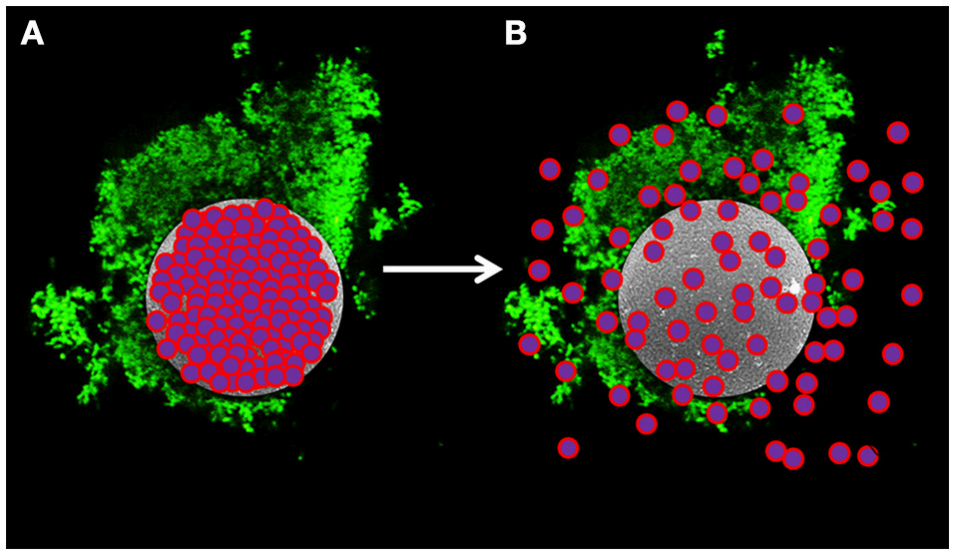
**Model of novel probiotic delivery formulation. (A)** A biofilm of *L. reuteri* (green) adhered to the surface of a dextranomer microsphere (gray) that contained beneficial compounds as cargo (purple and pink spheres) within the lumen of the microsphere. **(B)** Over time, the cargo will diffuse out of the porous microsphere thereby facilitating ready access by the adhered bacteria.

We have devised a novel delivery method that utilizes hollow semi-permeable, biocompatible and biodegradable microspheres comprised of cross-linked dextran (dextranomer microspheres or DMs). We have previously shown that allowing the probiotic bacterium *Lactobacillus reuteri* to adhere to DMs prior to oral delivery reduces the incidence of experimental necrotizing enterocolitis (NEC) (a disease of high mortality in premature infants born under 1,500 g, Neu and Walker, 2011) by 50% with a single dose in a rat model of the disease (Olson et al., 2016). Importantly, in the same study, a single dose of planktonic *L. reuteri* showed no prophylactic effect. We surmised that it was delivery of the probiotic bacteria in the biofilm state that facilitated the significantly increased therapeutic value of *L*. *reuteri* in this model system. According to our model (as shown in Figure 1), bacteria on the surface of the DMs in the form of a biofilm (i.e., an adhered community of bacteria that produces a self-forming protective matrix to resist adverse environmental conditions, Hall-Stoodley et al., 2004), would also have ready access to any beneficial compounds that diffused from the lumen of the DMs.

The choice to pair the Gram-positive bacterium *L. reuteri* with DMs is many fold. *L. reuteri* is a popular choice for commercialization due to its production of lactic acid and the antimicrobial compound reuterin, a metabolite of glycerol metabolism known chemically as 3-hydroxypropionaldehyde (3-HPA), which typically exists in equilibrium with other downstream products such as 3-HPA hydrate and acrolein (Talarico et al., 1988; Engels et al., 2016). Reuterin induces oxidative stress in a broad range of microorganisms (Schaefer et al., 2010), and is highly effective at inhibiting growth of many gastrointestinal pathogens (el-Ziney and Debevere, 1998; Arques et al., 2004; Spinler et al., 2008; De Weirdt et al., 2012). In addition to the antimicrobial reuterin, *L. reuteri* also produces anti-inflammatory factors including histamine that modulate cytokine production *in vitro* (Jones and Versalovic, 2009) and ameliorate the symptoms of inflammatory bowel disease (Ghouri et al., 2014). Indeed, in animal models, *L. reuteri* can prevent the exacerbating effects of the physiological stress response on colonic inflammation. However, once administration of *L. reuteri* is terminated, colonic inflammation is again exacerbated (Mackos et al., 2016). Thus, strategies to enhance the ability of *L. reuteri* to better persist in the GI tract may have more impactful therapeutic value.

Integral to our probiotic formulation strategy is *L. reuteri*'s extracellular glucosyltransferase (GTF) protein, which in the strain of *L. reuteri* used in this study (DSM 20016, containing GTFW encoded by *gtfW*) (Leemhuis et al., 2013; Bai et al., 2015) catalyzes the formation of exopolysaccharides of glucose (glucans) from its sole known substrate maltose. Importantly, GTF proteins typically have a glucan binding domain that recognizes its own produced exopolysaccharide (Monchois et al., 1999; Kralj et al., 2004). The GTF protein, its substrate, and resulting glucan product are highly strain-specific in *L. reuteri*; some are characterized as producing dextran (primarily α-1,6 linkages), mutan (primarily α-1,3 linkages), or the aptly named reuteran (primarily α-1,4 linkages) (Kralj et al., 2002, 2004). Cell aggregation, biofilm formation, and gut colonization are directly linked to the activity of GTFA in *L. reuteri* strain TMW1.106; inactivating *gtfA* significantly diminishes the ability of *L. reuteri* to aggregate, form biofilms, and colonize the GI tract *in vivo* (Walter et al., 2008).

Our novel approach was to choose DMs (a macroscopic porous microsphere that is sold commercially for size exclusion chromatography, Porath and Flodin, 1959) as a biocompatible surface so as to take advantage of *L. reuteri's* GTFW native ability to bind to this cross-linked dextran (Tieking et al., 2005; Schwab et al., 2007; Walter et al., 2008). Our strategy is based on concurrent research which shows that the highly similar GTFs of *Streptococccus mutans*, an oral pathogen that contributes to tooth decay, binds to DMs with high affinity (Mooser et al., 1985) and, as a consequence of GTF being cell-associated, results in strong binding of *S. mutans* to DMs (Mashburn-Warren et al., submitted). Here in we show that GTFW-dependent binding of *L. reuteri* to DMs results in: one, selectivity of binding to DMs and as a result better binding of *L. reuteri* to colonic epithelial cells; two, protection against low pH and three, the ability of *L. reuteri* to acquire the luminal contents of the DMs at sufficiently high concentrations to enhance *L. reuteri*'s probiotic effects.

## Materials and methods

### Strains and culturing conditions

Bacterial strains, plasmids and oligonucleotides used are listed in Table 1. *L. reuteri* (ATCC 23272) and *Lactobacillus rhamnosus* GG (ATCC 53103) were grown in MRS (de Man, Rogosa, Sharpe) medium (De Man et al., 1960) (BD, Franklin Lakes, NJ) for 16 h at 37°C, 5% CO_2_. *Salmonella typhi* (strain JSG698) and *Citrobacter rodentium* (ATCC 51459) were grown in Lysogeny broth (LB) at 37°C, 5% CO_2_. *Clostridium difficile* (strain R20291) was grown in degassed brain-heart infusion (BHI) medium (BD, Franklin Lakes, NJ) at 37°C in an anaerobic chamber (Thermo Forma Scientific, 1025 Anaerobic System, Hampton, NJ) established with an atmosphere of 5% H_2_, 85% N_2_, and 10% CO_2_. DLD-1 (ATCC CCL-221) human colonic cells were grown in RPMI medium supplemented with 10% fetal bovine serum at 37°C, 5% CO_2_. FHs 74 Int (ATCC CCL-241) human fetal small intestinal cells were grown in Hybri-Care medium (ATCC 46-X) supplemented with 30 ng/ml epidermal growth factor (EGF) and 10% fetal bovine serum at 37°C, 5% CO_2_. The *gtfW* deletion strain (LMW500) was constructed by insertion of a chloramphenicol resistance cassette (cat) into the *gtfW* open reading frame by allelic exchange as described previously (Mashburn-Warren et al., 2012). Briefly, 1kb fragments upstream and downstream of *gtfW* were amplified by PCR using oligos oSG1082-1083 and oSG1084-1085, followed by cloning into pFED760 (Mashburn-Warren et al., 2012) using NotI/SalI and SalI/XhoI restriction sites, respectively. The *cat* cassette was amplified from pEVP3 (Mashburn-Warren et al., 2012) using oligos LMW34-35, followed by cloning into pFED760 that contained the upstream and downstream fragments of *gtfW* using the SalI restriction site. The resulting *gtfW* knock-out construct plasmid (pWAR500) was then introduced into *L. reuteri* ATCC 23272 by electroporation. *L. reuteri* electrocompetent cells were prepared by growing 5 ml of culture in MRS at 37°C with 5% CO_2_ until OD_600nm_ of ~1.0. Cells were then pelleted and resuspended in 10 ml of sterile cold 0.5 M sucrose and 10% glycerol twice, followed by a final resuspension in100 μl sterile cold 0.5 M sucrose and 10% glycerol. To this resuspension 1 μg of pWAR500 was added and the cell/DNA mixture was placed into an ice cold 2 mm electroporation cuvette (BioRad, Hercules, CA). Cells were electroporated at 2500V, 25 μF and 400 Ω using a BioRad Gene Pulser Xcell (BioRad, Hercules, CA). Immediately after electroporation, cells were resuspended in 1 mL of MRS and incubated at 30°C for 2 h, followed by serial dilution and plating onto MRS agar containing 5 μg/ml chloramphenicol and incubated at 30°C. The mutant was selected and confirmed as previously described (Chang et al., 2011).

**Table 1:** **Bacterial strains, cell lines, plasmids, and oligos used in this study**.

**Bacterial strains**	**Description**	**Source/Reference**
*Lactobacillus reuteri*	Wild type (GTFW)	American Type
ATCC 23272		Culture Collection
LMW500	*L. reuteri* 23272 Δ*gtfW*; Cm^R^	This study
LMW501	*L. reuteri* 23272 + pWAR501 Cm^R^	This study
LMW502	*E. coli* ER2566 + pWAR502	This study
LMW503	*L. reuteri* 23272 + pWAR503 Cm^R^	This study
*Lactobacillus rhamnosus*	Wild type (non-dextran forming GTF)	G. Rajashekara
GG ATCC 53102		J.S. Gunn
*Salmonella enterica*	Wild type (non-dextran forming GTF)	
*serovar typhi* TY2 ATCC		
700931		
*Citrobacter rodentium*	Wild type (non-dextran forming GTF)	American Type
ATCC 51459		Culture Collection
*Clostridium difficile*	Wild type (non-dextran forming GTF)	J.K. Spinier
R20291 (BI/NAP1/027)		
**Human cell lines**	**Description**	**Source/Reference**
DLD-1 ATCC CCL-221	Human colonic epithelial cells (colorectal adenocarcinoma)	G.E. Besner
FHs 74 Int ATCC CCL-241	Human fetal small intestinal epithelial cells	G.E. Besner
**Plasmids**	**Description**	**Source/Reference**
pWAR500	pFED760 (Mashburn-Warren et al., 2012) derivative containing *cat* and DNA fragments flanking *gtfW* to create insertion mutant; see Materials and Methods; Cm^R^, Erm^R^	This study
pWAR501	pJC156 (Mashburn-Warren et al., 2012) derivative containing the promoter region of *gtfW* upstream of the click beetle luciferase; see Materials and Methods; Cm^R^	This study
pWAR502	pTXB1 derivative containing *gtfW* (with its stop codon); see Materials and Methods; Amp^R^	
pWAR503	pJC156 (Mashburn-Warren et al., 2012) derivative containing the promoter region of elongation factor Tu (EF-Tu) upstream of the click beetle luciferase; see Materials and Methods; Cm^R^	This study
**Oligos**	DNA sequence^*^	**Source/Reference**
oSG1082	GCGTG**GCGGCCGC**CATTATTTTCATGTAGTGTATT T	This study
oSG1083	GCGTG**GTCGAC**CTTTTTTATGTCCATAATCTATT	This study
oSG1084	GCGTG**GTCGAC**GAAAATATTTAATATGAAAATGA	This study
oSG1085	GCGTG**CTCGAG**CCAAGCACTATTTCACGAGAAT	This study
LMW34	GCGTG**GTCGAC**GATGAAAATTTGTTTGATTT	Mashburn-Warren et al., 2012
LMW35	GCGTG**GTCGAC**TTATAAAAGCCAGTCATTAG	Mashburn-Warren et al., 2012
oSG1102	GCGTG**CTCGAG**CAACAAGAGTATCAGGGTAAAGC	This study
oSG1103	GCGTG**GTCGAC**TCCTTCCCAATAGATGATTGATT	This study
oSG1067	GCGTG**GTCGAC**ATGGTAAAACGTGAAAAAAATGT	This study
oSG1068	**GCGGCCGC**TCCGCCAGCTTTTTCTAATAACT	This study
oSG1120	GCGTG**GCTAGC**ATGAACCTGCCAACAATTCCTAA	This study
oSG1126	GCGTG**GCTCTTCCGCA**TTAAATATTTTCTTGGTTT	This study
oSG1069	GCGTG**CTCGAG**CGCAACAAATACAGTTTCTAATA	This study
oSG1070	GCGTG**GTCGAC**AAACCTCCTGATAATTTACAAGT	This study

*Cm^R^, chloramphenicol resistant; Erm^R^, Erythromycin resistant; Amp^R^, Ampicillin resistant*.

* *Sequences in bold indicate restriction enzyme sequences*.

To estimate transcription from the *gtfW* promoter (P_gtfW_), the P_gtfW_-*CBluc* reporter plasmid was constructed by amplifying the promoter region 350 bp upstream of the *gtfW* start codon (including the native ribosome binding site) by PCR using oligos oSG1102-1103. The resulting DNA fragment was inserted into pJC156 using the XhoI/SalI restriction sites. The click beetle luciferase (CBluc) gene was amplified from the *Streptococcus mutans strain* ldhCBGSm (Merritt et al., 2016) using oligos oSG1067-1068 and inserted downstream of the *gtfW* promoter region in pJC156 using SalI/NotI restriction sites. The resulting reporter plasmid pWAR501 was transformed into *L. reuteri* 23272 as described above to create the reporter strain LMW501.

The *E. coli gtfW* overexpression strain (LMW 502) was created by amplifying the *L. reuteri gtfW* open reading frame (including the stop codon) using primers oSG1120-1126. The resulting DNA fragment was inserted into pTXB1 (New England BioLabs, Ipswich, MA) using NheI/SapI restriction sites. The resulting plasmid, pWAR502 was then transformed into the *E. coli* expression strain ER2566 (New England BioLabs, Ipswich, MA) and selected on LB agar containing 100 μg/ml ampicillin and confirmed by DNA sequencing. This strain allows the overexpression of tagless GTFW protein.

To produce a *L. reuteri* strain constitutively expressing click beetle luciferase, a reporter plasmid was constructed by amplifying the promoter region 250 bp upstream of the elongation factor Tu (EF-Tu) start codon (including the native ribosome binding site) by PCR using oligos oSG1069-1070. The resulting DNA fragment was inserted into pJC156 using the XhoI/SalI restriction sites. The click beetle luciferase (CBluc) gene was amplified from the *S. mutans strain* ldhCBGSm (Merritt et al., 2016) using oligos oSG1067-1068 and inserted downstream of the EF-Tu promoter region in pJC156 using SalI/NotI restriction sites. The resulting reporter plasmid pWAR503 was transformed into *L. reuteri* 23272 as described above to create LMW503.

### Microsphere preparation and application

Anhydrous dextranomer microspheres (DMs; Sephadex® G-25 Superfine) were purchased from GE Healthcare Life Sciences (Pittsburgh, PA). Anhydrous cellulose microspheres (CMs; Cellulobeads D50) were obtained from Kobo Products, Inc. (South Plainfield, NJ). Anhydrous microspheres were hydrated in growth medium or water at 50 mg/ml then autoclaved for 20 min. For conditions with microspheres that contained maltose, sucrose, fructose, or glucose only, microspheres previously autoclaved in water were removed from solution on a vacuum filter apparatus and approximately 50 mg were collected via sterile loop into 1ml of filter-sterilized 1M solution of the sugar (see Figure S1). The microsphere mixture was then vortexed vigorously and incubated for 24 h at room temperature to reach equilibrium.

For application with *L. reuteri*, microspheres loaded with water, 1M maltose, 1M sucrose, 1M glucose, or 1M fructose were removed from solution on a vacuum filter apparatus and collected via a 10 μl sterile loop. Approximately 5 mg of hydrated microspheres were then added to 1 ml of 2 × 10^9^ CFU *L. reuteri* from an overnight culture that had previously been pelleted by centrifugation at 3220 × g for 10 min, washed twice with sterile 0.9% saline, and resuspended in 1 ml sterile saline. For experiments involving eukaryotic cell lines, 2 × 10^9^ CFU of bacteria were resuspended in 1 ml RPMI instead of saline. For experiments with no microspheres but equivalent volume of cargo, 10 μl of cargo was added to 1 ml of bacteria either in sterile saline or RPMI. For all experiments, the bacteria and microsphere mixture were incubated together at room temperature for 30 min (unless otherwise stated) to facilitate bacterial adherence and biofilm formation on the microsphere surface prior to use in assays.

### Microsphere adherence assay

*L. reuteri* culture was grown and prepared as described above and incubated with microspheres filled with either: water, 1M maltose, 1M sucrose, 1M fructose, or 1M glucose. To examine bacterial adherence to the microspheres, 300 μl of bacteria (from an overnight culture containing ~2 × 10^9^ CFU) in sterile saline and 5 mg of microspheres were combined and incubated for 5 min in a Micro Bio-Spin column (BioRad, Hercules, CA) (see Figure S2). The columns were then centrifuged (100 × g) for 1 min. The flow-through was serially diluted and plated to calculate the total number of non-adhered bacteria, and this value was subtracted from the total number of starting bacteria to derive the total number of adhered bacteria. For all experiments, a control preparation that consisted of bacteria with no microspheres was used.

### Reporter assay

The reporter strain LMW501 was grown at 37°C with 5% CO_2_in MRS or MRS containing 3% glucose, sucrose, fructose, or maltose and optical densities (OD_600nm_) of the cultures were measured throughout growth using an Epoch Microplate Spectrophotometer (BioTek Instruments Inc., Winooski, VT). At the indicated times, 80 μl aliquots of the bacterial cultures were mixed with 20 μl 2 mM D-luciferin in 0.1M citrate buffer, pH 6.0 and placed in a Falcon white flat-bottom 96-well plate (Becton, Dickinson Labware, Franklin Lakes, NJ), followed by luminescence detection using a Veritas Microplate Luminometer (Turner BioSystems Inc., Sunnyvale, CA).

### GTF enzymatic assay

*S. mutans* was grown in Todd Hewitt Broth at 37°C with 5% CO_2_ until early log phase (OD_600nm_ ~0.3), *L. reuteri* WT and the Δ*gtfW* mutant were grown in MRS at 37°C with 5% CO_2_ until late log phase (OD_600 nm_ ~1.0) for optimal *gtf* expression, and the *E. coli gtfW* overexpression strain was grown in LB broth at 37°C shaking (200 rpm) until mid-log phase (OD_600 nm_ ~0.4) followed by the addition of 1 mM IPTG to induce *gtfW* expression and was then grown at 37°C shaking for an additional 2 h. Whole cells of *S. mutans, L. reuteri* WT, *L. reuteri* Δ*gtfW*, and the *E. coli gtfW* overexpression strain were assayed for GTF activity as previously described (Bai et al., 2015) using Periodic acid-Schiff staining of SDS-PAGE gels.

### Cargo diffusion assay

The rate of cargo diffusion out of the microspheres was determined by tracking crystal violet, a small molecular weight dye (407.979 g/mol) (Fisher Scientific, Hampton, NJ). The microspheres were loaded with a 0.1% solution of crystal violet by incubating 20 mg of microspheres in 1 ml of 0.1% crystal violet solution either with or without added glycerol (40 or 80% v/v) overnight to reduce the diffusion rate by increasing viscosity. After 16 h, excess crystal violet solution was removed from the microspheres as described above using a vacuum filter apparatus. The crystal violet-loaded microspheres were then placed into 1 ml of water, and aliquots of water were removed and analyzed for diffusion of crystal violet into solution using an Epoch Microplate Spectrophotometer (BioTek, Winooski, VT) at OD_590nm_ every hour for 16 h. Percent diffusion was calculated using the equivalent amount of crystal violet within the microspheres (10 μl) in water as a control equivalent to 100% cargo diffusion.

### Reuterin assay

Production of reuterin by *L. reuteri* was measured via a quantitative colorimetric assay (Cadieux et al., 2008). As this assay did not differentiate between similar aldehyde products, measurements included 3-HPA and any potential derivatives, such as acrolein and 3-HPA hydrate. *L. reuteri* was grown overnight in MRS as described above, 1 ml aliquots of 2 × 10^9^ CFU were pelleted at 3,220 × g for 10 min, washed twice with sterile saline, and resuspended in either 1 ml of sterile saline or 1 ml sterile saline containing 2% v/v glycerol. DM containing 0, 2, 10, 20, 30, 40, 50, 60, 70, or 80% glycerol were prepared as described above for other cargo, and added to the resuspended *L. reuteri* in saline (so that the only source of glycerol available for reuterin production was via the microsphere cargo) for 1 h at 37°C. Cells were then pelleted again and the reuterin-containing supernatant was removed, filtered through a 0.45 μm filter, and assayed for reuterin as described in Cadieux et al. (2008) without modification. A standard curve using reuterin at known concentrations was used to extrapolate bacterial-produced reuterin concentrations from DM-glycerol and the 2% v/v glycerol control experimental conditions.

### *L. reuteri* survival with DM-80% glycerol

Overnight cultures of WT *L. reuteri* were aliquoted into microcentrifuge tubes, centrifuged, washed twice with sterile saline, and resuspended in either 1 ml saline or 1 ml MRS medium. Five mg of either DM-water or DM-80% glycerol were then added to the tubes and incubated at 37°C. At hourly intervals the tubes were mixed thoroughly and aliquots were taken for subsequent serial dilution and plating for viable CFU of bacteria.

### Histamine assay

Production of histamine from L-histidine by *L. reuteri* was measured via ELISA (Enzo Life Sciences, Inc., Farmingdale, NY). *L. reuteri* was grown overnight in MRS as described above, 1 ml aliquots of 2 × 10^9^ CFU were pelleted at 3220 × g for 10 min, washed twice with sterile saline, and resuspended in one of the following conditions: sterile saline, saline with 3% maltose, saline with 2% v/v glycerol, 4 mg/ml L-histidine (Sigma-Aldrich, St. Louis, MO), 4 mg/ml L-histidine with 3% maltose, or 4 mg/ml L-histidine with 2% v/v glycerol. 5 mg of DM containing either 4 mg/ml or 30 mg/ml L-histidine were added to media lacking L-histidine, so that the only source of L-histidine for *L. reuteri* was as cargo diffusing out of the DMs. Each condition was then incubated at 37°C for 2 h, after which time the contents were pelleted and the supernatant was removed for histamine quantification via a histamine ELISA kit (Enzo Life Sciences, Inc., Farmingdale, NY) following the manufacturer's instructions without modifications. All conditions were done in at least triplicate.

### pH survivability assay

Bacteria were exposed to a synthetic gastric acid equivalent to determine survival at pH 2. Gastric acid equivalent is a modified version of synthetic gastric fluid (Cotter et al., 2001), composed of 0.1M HCl, 0.1M NaCl, and 0.01M KCl, with pH adjusted to 2 using 0.1M NaOH. For the assay, 1 ml of 2 × 10^9^ CFU of *L. reuteri* from a fresh overnight culture were pelleted at 3,220 × g for 10 min, washed twice with sterile saline, and resuspended in 1 ml 0.9% sterile saline. The cells were incubated for 30 min with approximately 5 mg of loaded or unloaded microspheres as described above, and the bacteria-microsphere mixture was diluted 1:100 directly into gastric acid equivalent. Aliquots of the inoculated acid solution were mixed, serially diluted, and plated at hourly time points for 4 h to determine the number of viable bacteria. Bacteria without microspheres were used as a control.

### Adherence to intestinal epithelial cells

DLD-1 colonic cells and FHs 74 small intestinal cells were cultured as described above. When the adherent epithelial cells reached confluence, the growth medium was removed, cells were washed twice with sterile phosphate buffered saline (PBS), and trypsin-EDTA (0.25%) was added for 10 min at 37°C to dislodge the cells from the culture flask surface. Total epithelial cells were counted using a hemacytometer (Hausser Scientific, Horsham, PA). Cells were then diluted to a concentration of 5 × 10^5^ cells/ml and 1 ml per well was seeded into a 24-well plate and incubated at 37°C, 5% CO_2_. After either 48 h (for DLD-1 cells) or 120 h (for FHs 74 cells) of growth, the spent medium was removed and replaced with 1 ml of RPMI or Hybri-Care medium containing 2 × 10^9^ CFU of *L. reuteri* alone, *L. reuteri* with 5 mg water-filled DMs, *L. reuteri* with 5 mg sucrose-filled DMs, or *L. reuteri* with 5 mg maltose-filled DMs. After a 1 h incubation, the spent medium was removed and the well was washed with 1 ml of sterile PBS 3 times to remove non-adhered bacteria. The remaining epithelial cells, with adhered bacteria, were then trypsinized as described above, serially diluted, and plated onto solid MRS medium for enumeration of total adhered bacteria. For confocal microscopy experiments with DLD-1, Nunc Lab-Tek 8-well borosilicate chamber slides (Fisher Scientific, Hampton, NJ) were used in place of 24-well plates. The chamber slides were treated with collagen prior to DLD-1 seeding to improve cellular adherence using the following protocol: a mixture of 100 μl of 7.5% BSA (Sigma-Aldrich, St. Louis, MO), 50 μl of 3.79 mg/ml collagen (Millipore, Temecula, CA), 100 μl of 1 mg/ml rat fibronectin (Biomedical Technologies, Stoughton, MA), and 9.75 ml of PBS was prepared, and 200 μl of this solution was added per chamber slide well. After incubation for 1 h at 37°C, the solution was removed from the well, and epithelial cells were seeded and grown as described above.

### Mucin adherence assay

Mucin agar plates were created using porcine stomach mucin (Sigma-Aldrich, St. Louis, MO). Mucin agar plates contained 2% mucin and 0.8% agar to simulate the consistency of the mucus layer found *in vivo* (Macfarlane et al., 2005; Van den Abbeele et al., 2009). To assess *L. reuteri*'s ability to bind mucin, 2 × 10^9^ CFU of *L. reuteri* that contained a plasmid that encoded expression of the click beetle luciferase enzyme either planktonically or bound to 5 mg DM-water, DM-sucrose, or DM-maltose were incubated on both mucin agar and agar without mucin stationary at room temperature. After 60 min, the non-adhered *L. reuteri* were removed by washing the plates twice with sterile saline. The luciferase substrate D-luciferin (Sigma-Aldrich, St. Louis, MO) was then added to the plates at a concentration of 0.4 mM to visualize the remaining adhered bacteria. Relative luminosity generated from the bacteria on the plates was measured using a FluorChem E system (ProteinSimple, San Jose, CA) with a 20 min exposure setting. To assess the number of bacteria bound to the mucin within the plate (and not any background binding that may occur to the agar within the plate), the amount of luminescent signal from the agar-only plates was subtracted from the mucin agar plates.

### Confocal microscopy

All confocal laser scanning microscopy (CLSM) was performed using a Zeiss LSM 510 confocal microscope (Ziess AG, Oberkochen, Germany). For fluorescent staining, dextranomer and cellulose microspheres were pre-stained with Congo Red (Fisher Scientific, Hampton, NJ) prior to incubation with the cargo (e.g., sucrose) and experiments with bacteria. *L. reuteri* was stained with SYTO 9 (Life Technologies, Carlsbad, CA). Differential fluorescent visualization was performed using the following settings: Congo Red excitation 554 nm/emission 568 nm, and SYTO 9 excitation 490 nm/emission 525 nm. Samples were fixed using a custom biofilm fixative containing 1.5% paraformaldehyde, 0.025% glutaraldehyde, 4.0% acetic acid, and 0.1M phosphate buffer at pH 7.4 (Devaraj et al., 2015). All microscopy was performed on samples in Nunc Lab-Tek 8-well borosilicate chamber slides (Fisher Scientific, Hampton, NJ). For CLSM experiments with DLD-1 epithelial cells, DLD-1 was stained with 4′,6-Diamidino-2-Phenylindole (DAPI, Life Technologies, Carlsbad, CA), *L. reuteri* was stained with carboxyfluorescein succinimidyl ester (CFSE, Life Technologies, Carlsbad, CA). AxioVision software (Ziess AG, Oberkochen, Germany) and ICY (de Chaumont et al., 2012) were used to analyze images and create figures from CLSM images. COMSTAT (Heydorn et al., 2000) software was used to quantify bacterial biomass in CLSM images.

For *in vitro* biofilm assays, overnight cultures of WT and Δ*gtfW L. reuteri* were diluted into fresh MRS growth medium to 0.01 OD_600nm_, incubated at 37°C 5% CO_2_ for 2.5 h until reaching 0.65 OD_600 nm_, diluted 1:2,500 into either MRS, MRS + 3% sucrose, or MRS + 3% maltose, seeded into 8-well borosilicate chamber slides and incubated for 1, 3, or 6 h at 37°C 5% CO_2_. At the designated time intervals, the bacteria were stained for viability with LIVE/DEAD stain, fixed, visualized via confocal microscopy, and quantified via COMSTAT analysis of the fluorescent signal.

### Scanning electron microscopy

All scanning electron microscopy (SEM) was performed using a Hitachi S-4800 field emission SEM (Hitachi, Tokyo, Japan). Samples were prepared as described in “Adherence to colonic cells,” with the exception that DLD-1 human colonic epithelial cells were grown on 15 mm diameter thermanox coverslips (Electron Microscopy Sciences, Hatfield, PA) placed within the well of a 12-well plate. Samples of DLD-1 cells and adhered bacteria were fixed overnight at 4°C in a solution of 2.5% glutaraldehyde in 0.1M phosphate buffer (pH 7.2). Samples were then washed with double distilled water and stained with a 1% solution of osmium tetroxide (Sigma-Aldrich, St. Louis, MO) in 0.1M phosphate buffer (pH 7.2) for 1 h, washed for 5 min, stained with a 1% solution of thiocarbohydrazide (Sigma-Aldrich, St. Louis, MO), washed for 5 min, and further stained with 1% osmium tetroxide for 30 min. Samples were then dehydrated using a graded series of ethanol: 25% ethanol for 15 min, 50% ethanol for 15 min, 70% ethanol for 30 min, 95% ethanol for 15 min (twice), 100% ethanol (twice), a 1:1 mixture of 100% ethanol to 100% hexamethyldisilazane (HMDS, Sigma-Aldrich, St. Louis, MO) for 100 min, 100% HMDS for 15 min, and a final immersion in 100% HMDS that was allowed to air dry overnight. Dehydrated sample coverslips were then mounted onto 15 mm diameter metal SEM specimen stubs (Electron Microscopy Sciences, Hatfield, PA) using colloidal silver (Electron Microscopy Sciences, Hatfield, PA). The outer edge, where the stub and coverslip meet, was then coated with a light layer of colloidal silver, and allowed to dry overnight. Samples were sputter coated with gold and palladium for 2 min at 25 mA using an Emitech K550X sputter coater (Quorum Technologies Ltd., Laughton, United Kingdom).

### Statistical analysis

All experiments were conducted a minimum of three times and statistical analysis was performed via a Student's *t*-test using GraphPad Prism software (GraphPad Software, Inc., La Jolla, CA), wherein a *P*-value less than 0.05 was accepted as significant.

## Results

### Maltose or sucrose within the lumen of DMs improved *L. reuteri* adherence to DMs in a GTF-dependent manner

**Figure 2:**
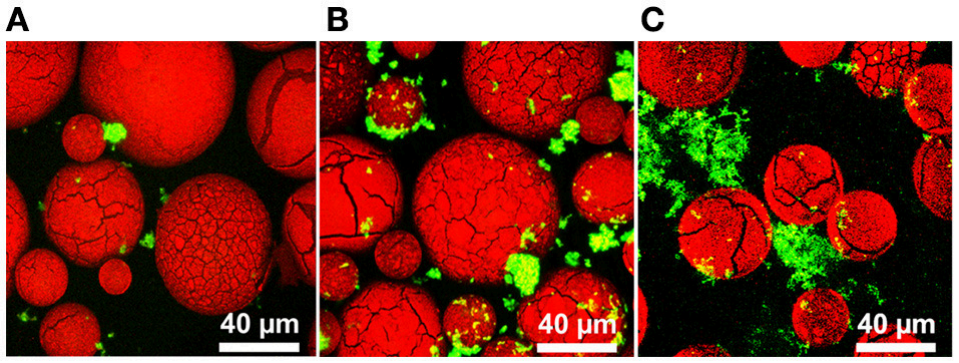
*****L. reuteri*** binds to dextranomer microspheres**. Confocal laser scanning microscopy (CLSM) of *L. reuteri* adhered to DMs. **(A)** Water-filled DMs, **(B)** sucrose-filled DMs, and **(C)** maltose-filled DMs after incubation with *L. reuteri* for 30 min showed that *L. reuteri* adherence to DMs can be enhanced to incorporate biofilm-promoting cargo within the DM lumen (green: bacteria stained with SYTO 9, red: DMs stained with Congo Red).

Our strategy was to have probiotic bacteria adhere to a biocompatible surface to induce the formation of a biofilm (Figure 1). To investigate this, we differentially stained DMs with Congo Red and *L. reuteri* with SYTO 9, and examined binding via confocal laser scanning microscopy (CLSM). As shown in Figure 2, aggregates of bacteria were associated with the surface of numerous DMs which indicated that *L. reuteri* was able to adhere to the DM surface within the time allotted. Since DMs are cross-linked glucan similar to the native reuteran produced by *L. reuteri*, we hypothesized that either an increase in GTFW (for enhanced binding to DMs) or production of glucan to stimulate aggregation and biofilm formation would facilitate the adhered state of *L. reuteri*. To this end, we compared adherence of *L. reuteri* to DMs that contained luminal cargo of either sucrose (an inducer of *gtfW* expression but not a substrate for GTFW; see Figure S3) or maltose (the sole substrate of GTFW). As shown in Figures 2B,C, compared to DMs that contained only water within the lumen (Figure 2A) there were greater numbers of *L. reuteri* adhered to DMs with either sugar as cargo.

**Figure 3:**
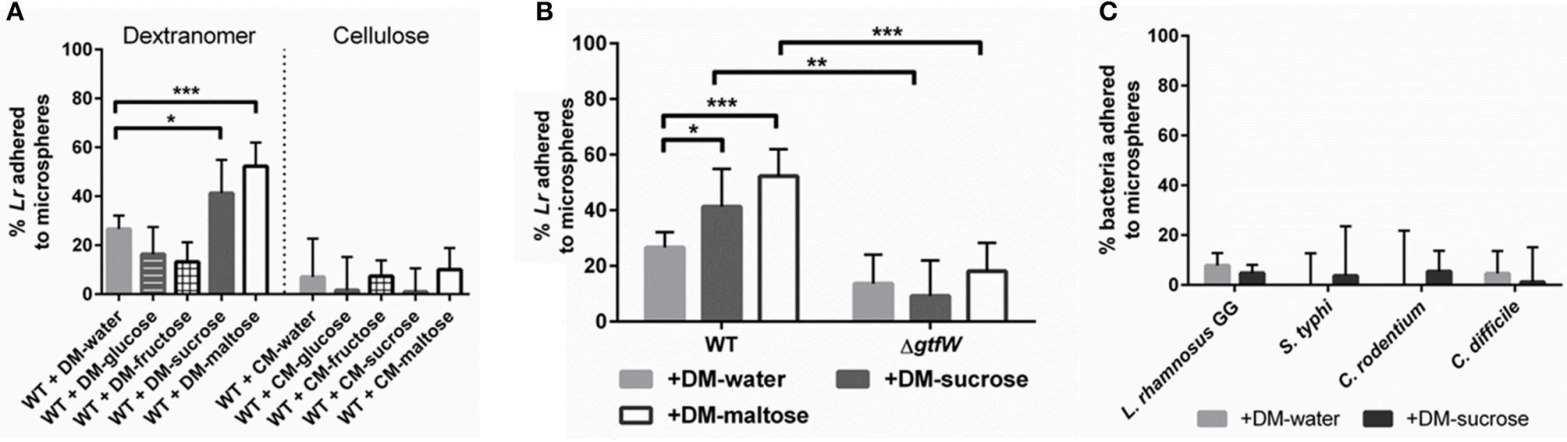
**Microsphere composition and lumen cargo affected ***L. reuteri*** adherence, ***L. reuteri*** adhered to DMs in GTFW-dependent manner, and bacteria lacking GTF did not bind to DMs**. A spin column assay was performed to assess relative bacterial adherence to microspheres. Bacteria were incubated for 5 min with 5 mg of microspheres, centrifuged at 100 × g to separate bound and unbound bacteria, then CFU of non-adhered bacteria were quantified in the flow-through of the spin column. **(A)** Microspheres composed of either cross-linked dextran (DM) or cross-linked cellulose (CM) were filled with water or various sugars at a concentration of 1M to determine which microsphere type supported greatest adherence of *L. reuteri*. **(B)** Relative WT and Δ*gtfW L. reuteri* adherence to DM showed that *L. reuteri* adhered to DMs in a GTF-dependent manner. **(C)** Non-GTF expressing bacteria were similarly tested for microsphere adherence with water-loaded and sucrose-loaded DMs. Error bars represent standard error of the mean. Statistical significance is indicated by the following: ^*^P < 0.05, ^**^P < 0.01, ^***^P < 0.0005.

To further investigate *L. reuteri*'s ability to bind DMs, we tested other DM lumen compounds that we hypothesized should not affect GTFW protein mediated binding and thus unlikely to support increased adherence to DMs. For this assay we chose the monosaccharide subunits of maltose and sucrose (e.g., glucose for maltose, glucose and fructose for sucrose), which the GTF enzyme cannot utilize to catalyze glucan polymers. Interestingly, fructose (and not glucose) was shown to induce *gtfW* expression at a rate similar to sucrose, but did not result in enhanced binding to DMs as was found with sucrose (Figure S3A, Figure 3A).

To determine if this GTFW-dependent binding is specific to the glycosyl linkages of DMs, we compared *L. reuteri* binding to cellulose microspheres (CMs), as DMs are composed of polymers of glucose with α-linkages while CMs possess β-linkages between the glucose units (Updegraff, 1969; Kralj et al., 2002). As shown in Figure 3A, only ~10% of *L. reuteri* adhered to CMs regardless of luminal contents. Collectively the data in Figure 3 indicated that *L. reuteri* does not bind to CMs, binding to DMs was GTFW-dependent and further, that inclusion of maltose or sucrose significantly enhanced the binding of *L reuteri* to DMs. We hypothesized that the predicted glucan binding domain of GTFW is a necessary component of *L. reuteri's* ability to adhere to DMs. To further test if the adherence to DM is GTF-dependent, we created a mutant strain of *L. reuteri* (LMW500) with a chloramphenicol resistance gene inserted in place of the *gtfW* gene. As shown in Figure 3B, the Δ*gtfW* strain was not able to bind to DMs as effectively as the wild type (WT) in our spin column assay, regardless of the cargo within the DM lumen. To further demonstrate the difference between the WT and Δ*gtfW*, we examined biofilm formation on glass chamber slides in media supplemented with sucrose or maltose (Figure S4). After a 1 h incubation, the WT had more bacteria present and noticeably more bacterial aggregation when sucrose or maltose was added to the growth medium (Figures S4A,B). After 3 and 6 h with sucrose or maltose supplemented media, the WT displayed a significantly more robust biofilm with greater biomass compared to the *gtfW* mutant under every condition, with significantly more cells present when sucrose or maltose was in the growth medium (Figures S4A,C,D).

We next tested whether bacteria that do not express a similar GTF would lack the adherent phenotype shown in Figures 3A,B. To examine this, we performed our DM adherence assay with another probiotic bacterium and three enteric pathogens that *L. reuteri* would likely encounter within the gastrointestinal tract: *Lactobacillus rhamnosus* GG, a Gram-positive bacterium commonly found in the genitourinary system and sold commercially as a probiotic; *Salmonella typhi*, a Gram-negative bacterium responsible for typhoid fever in humans; *Citrobacter rodentium*, a Gram-negative bacterium that causes colitis in rodents; and *Clostridium difficile*, a Gram-positive spore-forming bacterium that can cause severe colitis and recurring infections in humans. As shown in Figure 3C, all of the non-GTF expressing bacteria showed minimal adherence to DMs, regardless of cargo present within the DM lumen.

### Diffusion of cargo from DMs

**Figure 4:**
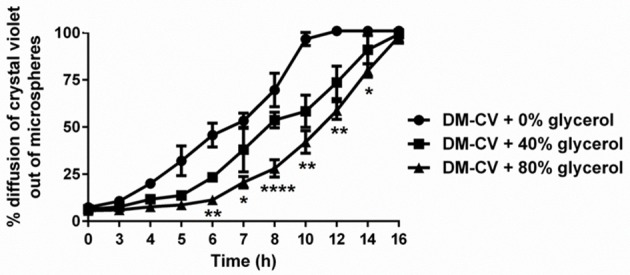
**Diffusion of cargo out of microspheres over time**. Crystal violet (CV)-loaded DMs with and without glycerol (added to increase viscosity) were assayed to determine the relative rate of CV diffusion from the microspheres. With 0% added glycerol, CV diffused at a higher rate (100% diffusion after 10 h) compared to DMs that contained 40 or 80% glycerol. We observed 100% diffusion from DMs after 16 h regardless of viscosity. Error bars represent standard error of the mean. Statistical significance from DMs with 0% added glycerol is indicated by the following: ^*^P < 0.05, ^**^P < 0.01, ^****^P < 0.0001.

Initial binding of bacteria to DMs is a critical component of our formulation, however equally important is the ability to co-deliver beneficial luminal cargo needed by the adherent bacteria during transit of DMs through the gastrointestinal tract. Targeted delivery of maltose (or any other beneficial compound) via diffusion out of the DMs directly to the probiotic bacterium over time was a desired feature of our system (Figure 1). However, since the method of cargo delivery would be diffusion through the porous surface of the microsphere and not its degradation, such as occurs in poly(lactic-co-glycolic) acid (PLGA) microspheres (Danhier et al., 2012), the rate of diffusion is dependent upon the size of the microsphere, the mass of the solute, and the viscosity of the diluent. As proof of concept, we filled the DMs with crystal violet, a small molecular weight stain (407.979 g/mol), and tested the diffusion rate of the dye out of the DMs with and without changing the viscosity of the solution in the DM lumen. As shown in Figure 4, the crystal violet diffused out of the DM lumen with a half-life of ~6 h. When the viscosity was increased by adding 40% glycerol, the half-life of release was increased to ~8 h. At 80% glycerol, the half-life of crystal violet release was further enhanced to 12 h. By 16 h >95% of all of the crystal violet had been released under all tested conditions.

### *L. reuteri* produced reuterin from glycerol-loaded microspheres

An important feature of *L. reuteri*'s function as a probiotic bacterium is its ability to compete with pathogenic bacteria within the host potentially via production of antimicrobials e.g., extracellular reuterin (Cleusix et al., 2007; Spinler et al., 2008). Due to limited glycerol availability, suboptimal endogenous concentrations of glycerol in the GI tract would likely limit adequate reuterin production. In order to obviate the need to provide high levels of glycerol to satisfy *L. reuteri*'s optimal needs, we provided targeted delivery of glycerol directly to the bacteria attached to the surface of DMs. To test this *in vitro*, we utilized a colorimetric assay for reuterin production (Cadieux et al., 2008). As shown in Figure S5, DMs filled with glycerol concentrations ranging from 10 to 80% were able to induce reuterin production. Compared to the 2% glycerol solution control, DMs filled with 80% glycerol produced on average 53% more reuterin in 1 h (average concentration of reuterin produced: 2% glycerol = 40 mM, DM-80% glycerol = 61 mM). To determine if the 80% glycerol or the resulting reuterin/downstream metabolites of glycerol fermentation produced by *L. reuteri* is toxic to *L. reuteri*, we compared hourly colony forming units (CFU) of *L. reuteri* incubated with either DM-water or DM-80% glycerol, in either sterile saline or MRS growth medium. As shown in Figure S6, there was no loss of CFU regardless of DM cargo when *L. reuteri* was incubated in MRS. Incubating *L. reuteri* in saline did result in a steady loss of viable CFU over time, though there was no difference in viability between the DM-water and DM-80% glycerol over this time, suggesting the loss of CFU was not due to any potentially toxic compounds, such as reuterin or acrolein, from glycerol fermentation (Figure S6). As acrolein in particular is known to be toxic to humans and is a byproduct of reuterin production, we next calculated the maximum possible amount of acrolein that could be produced from the dosage of *L. reuteri* and volume of glycerol provided via DMs in our formulation, assuming all available glycerol was converted 1:1 into acrolein. As shown in Figure S7, the amount of acrolein that could possibly be produced via our formulation is a nominal ~6 μg (for reference, the World Health Organization recommends less than 7.5 μg/kg body weight per day) (Gomes et al., 2002). From these results and the data presented in Figure 4, we hypothesized that DMs loaded with glycerol would have two beneficial effects *in vivo*, namely slowing the release of beneficial cargo and providing a substrate for reuterin production.

### *L. reuteri* produced histamine from L-histidine-loaded microspheres

**Figure 5:**
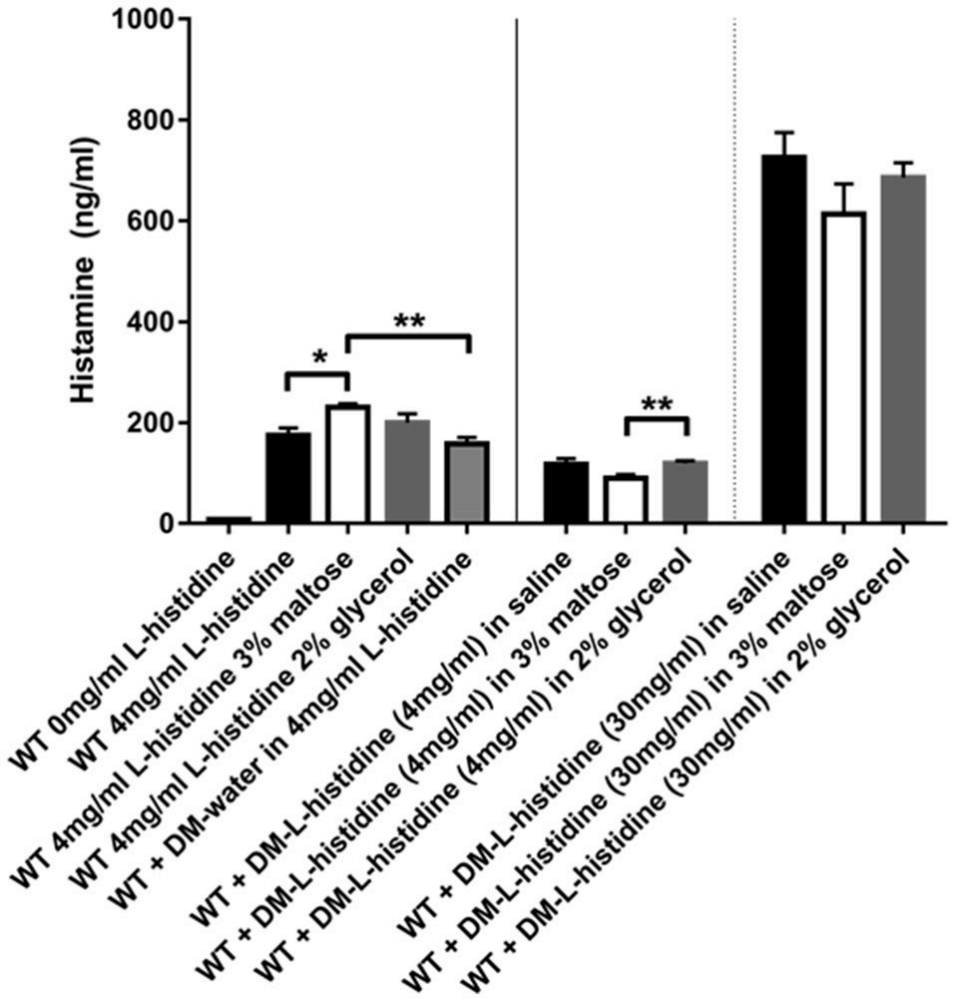
**Histamine can be produced by ***L. reuteri*** from L-histidine delivered via DM**. Stationary phase WT *L. reuteri* was incubated for 2 h in either saline with and without 3% maltose or 2% glycerol, or 4 mg/ml L-histidine with and without 3% maltose or 2% glycerol. Histamine production was increased with addition of 3% maltose to 4 mg/ml L-histidine solution (white bar black border) compared to just 4 mg/ml L-histidine (black bar and gray bar black border). When L-histidine at 4 mg/ml was provided via DM the overall levels of histamine produced were significantly lower (middle 3 bars) compared to L-histidine provided in solution (left 4 bars), likely due to less immediate availability of L-histidine to the bacteria. However, when the concentration of L-histidine loaded into DM was increased to 30 mg/ml, significantly more histamine was produced (right 3 bars) despite any caveats related to slower access to L-histidine due to availability only via diffusion out of DM. Error bars represent standard error of the mean. Statistical significance is indicated by the following: ^*^P < 0.05, ^**^P < 0.01.

Histamine produced by *L. reuteri* has previously been shown to inhibit pro-inflammatory cytokines such as TNF via H_2_ receptors and reduce colitis in an animal model (Thomas et al., 2012; Gao et al., 2015). Our microsphere-based approach provides a unique method for delivery of the histamine precursor substrate L-histidine to *L. reuteri*. To test this *in vitro*, we filled DMs with 30 mg/ml and 4 mg/ml L-histidine and measured the amount of histamine produced by the bacteria when the only source of L-histidine was via diffusion out of the DMs. As shown in Figure 5, DM- L-histidine (4 mg/ml) resulted in histamine levels only slightly lower than those produced when bacteria were incubated in 4 mg/ml L-histidine solution without DMs. When the DMs were loaded with a higher concentration of L-histidine, the amount of histamine produced was 6–7 times greater than the lower 4 mg/ml concentration, consistent with the DM-L-histidine (30 mg/ml) providing ~7 times more L-histidine than the DM-L-histidine (4 mg/ml) (Figure 5). In addition, we tested whether other cargo relevant DM cargo substrates, such as maltose and glycerol, would negatively affect histamine production. Addition of glycerol did not result in reduced histamine production, regardless of whether the L-histidine was in solution or provided via DMs (Figure 5). With addition of maltose, histamine production actually increased when L-histidine was provided in solution, but statistically unchanged when L-histidine was provided via DMs (Figure 5).

### Microspheres filled with sucrose or maltose improved *L. reuteri* survival at low pH

**Figure 6:**
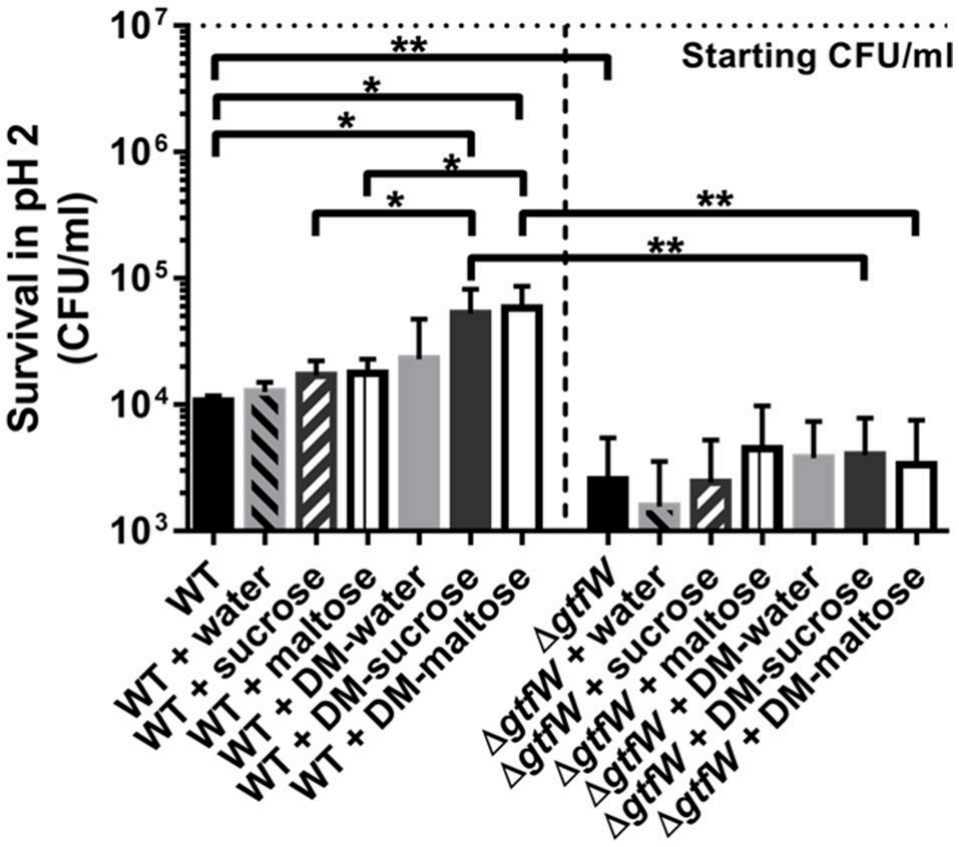
**Gastric acid survival**. WT and Δ*gtfW L. reuteri* (10^7^ CFU/ml) viability after 4 h in pH 2 synthetic gastric acid in the absence or presence of 5 mg of DMs that contained water, sucrose (1M), or maltose (1M) as cargo, or 10 μl of the cargo alone without DMs. Relative survival in acid was enhanced when WT *L. reuteri* was adhered as a biofilm on DMs that contained sucrose or maltose compared to equivalent volumes of the same cargo delivered without DMs, which indicated that the biofilm phenotype contributed to better survival during exposure to low pH. Δ*gtfW* showed decreased resistance to acid compared to the WT, regardless of the presence or absence of either DMs or sugar alone. Error bars represent standard error of the mean. Statistical significance is indicated by the following: ^*^P < 0.05, ^**^P < 0.01.

Orally consumed probiotics face a significant pH challenge upon reaching the stomach, where pH values are as low as 1.5 when the stomach is empty (Dressman et al., 1990). Enhancing the ability to deliver a maximal number of viable *L. reuteri* to the colon is crucial to its sustainability and effectiveness as a probiotic. We thereby hypothesized that *L. reuteri* bound to the surface of DMs in the form of a biofilm would increase survival upon exposure to acid, and that DMs filled with sucrose or maltose would result in even greater survival in a GTFW-dependent manner. As shown in Figure 6, less than 0.1% of WT *L. reuteri* without DMs survived in synthetic gastric acid after 4 h at pH 2, which resulted in a nearly 3 log loss of viable probiotic. Addition of water-filled DMs did not significantly alter the survival rate of WT *L. reuteri* in gastric acid; however, when either DM-sucrose or DM-maltose was delivered with WT, nearly 1 log more survived the acid stress (Figure 6). To show that the protective effect is dependent on the microspheres and not the cargo within the DM lumen, we also incubated *L. reuteri* with the equivalent amount of diffusible cargo without the DMs. Acid survival in the presence of cargo only was no different than *L. reuteri* alone (Figure 6), which strongly indicated the importance of the bacterial biofilm-on-DM delivery system for the observed protective effect.

To investigate whether this phenotype is GTFW-dependent, we also tested synthetic gastric acid survival using the Δ*gtfW* strain of *L. reuteri* and found that the beneficial effect of DM-sucrose and DM-maltose was lost (Figure 6). Interestingly, the mutant also showed deficiency in acid survival without DMs compared to WT, which indicated that GTFW's role in cellular aggregation and biofilm formation (Figure S4) may contribute significantly to survival in synthetic gastric acid.

### Microspheres promote *L. reuteri* adherence to human intestinal epithelial cells

**Figure 7:**
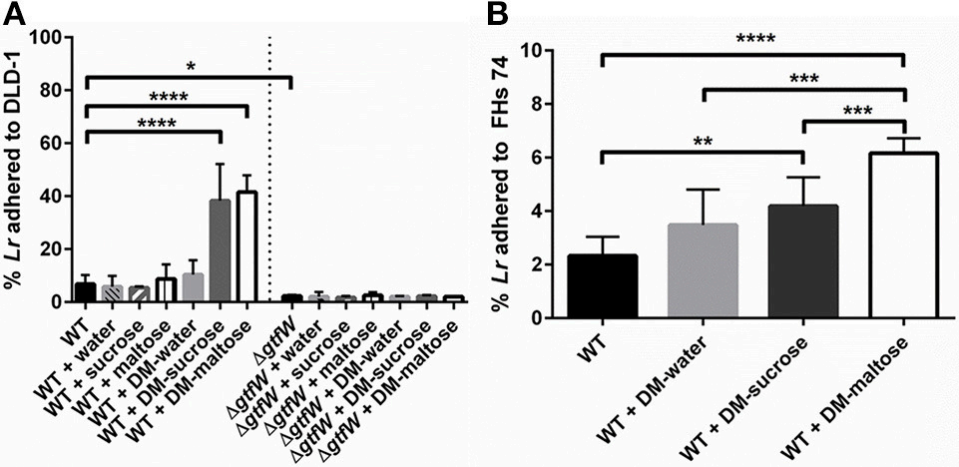
**Delivery of ***L. reuteri*** adhered to DMs as a biofilm supported increased adherence to intestinal epithelial cells. (A)** *L. reuteri* WT and Δ*gtfW* adhered as a biofilm on DMs that contained either water, sucrose (1M), or maltose (1M), or the equivalent volume of sugar alone (without DMs), were examined for relative adherence to human colonic DLD-1 cells after incubation for 60 min. Significantly more WT adhered to DLD-1 cells when delivered as a biofilm on the surface of DMs that contained sucrose or maltose, compared to water-filled DMs or the equivalent volume of sugar alone. Significantly fewer Δ*gtfW* mutant cells adhered to DLD-1 cells, regardless of cargo, which indicated that the GTFW protein contributes to *L. reuteri* adherence. **(B)** Adherence of WT to fetal small intestinal FHs 74 cells after 60 min incubation showed that providing *L. reuteri* adherent on the DM surface as a biofilm with either sucrose or maltose as cargo resulted in greater adherence to intestinal cells. Error bars represent standard error of the mean. Statistical significance is indicated by the following: ^*^P < 0.05, ^**^P < 0.01, ^***^P < 0.001, ^****^P < 0.0001.

Next, we examined what effect the DMs, the DM luminal cargo and the product of the *gtfW* gene have on the relative adherence of *L. reuteri* when delivered as planktonic cells or as biofilms on DMs to the human intestinal cell lines DLD-1 (adult human colonic epithelial cells) and FHs 74 Int (3–4 months gestation, small intestine epithelial cells) *in vitro*. As shown in Figure 7A, after a 1 h incubation on DLD-1 cells, significantly more WT *L. reuteri* (without DMs) adhered to the colonic cells compared to Δ*gtfW* either with or without DMs, which indicated that GTFW contributed to host cell adherence. When *L. reuteri* adhered to DMs that contained sucrose or maltose were added to colonic cells, relative adherence of WT *L. reuteri* to the colonic cells was increased by 4.7-fold for DMs that contained sucrose and by 5.2-fold for DMs that contained maltose (Figure 7A). Although overall fewer WT *L. reuteri* adhered to the FHs 74 cells than to DLD-1 cells, delivering the bacteria with either DM-sucrose or DM-maltose resulted in 1.8-fold (DM-sucrose) or 2.7-fold (DM-maltose) more adhered bacteria compared to WT bacteria without DM (Figure 7B).

**Figure 8:**
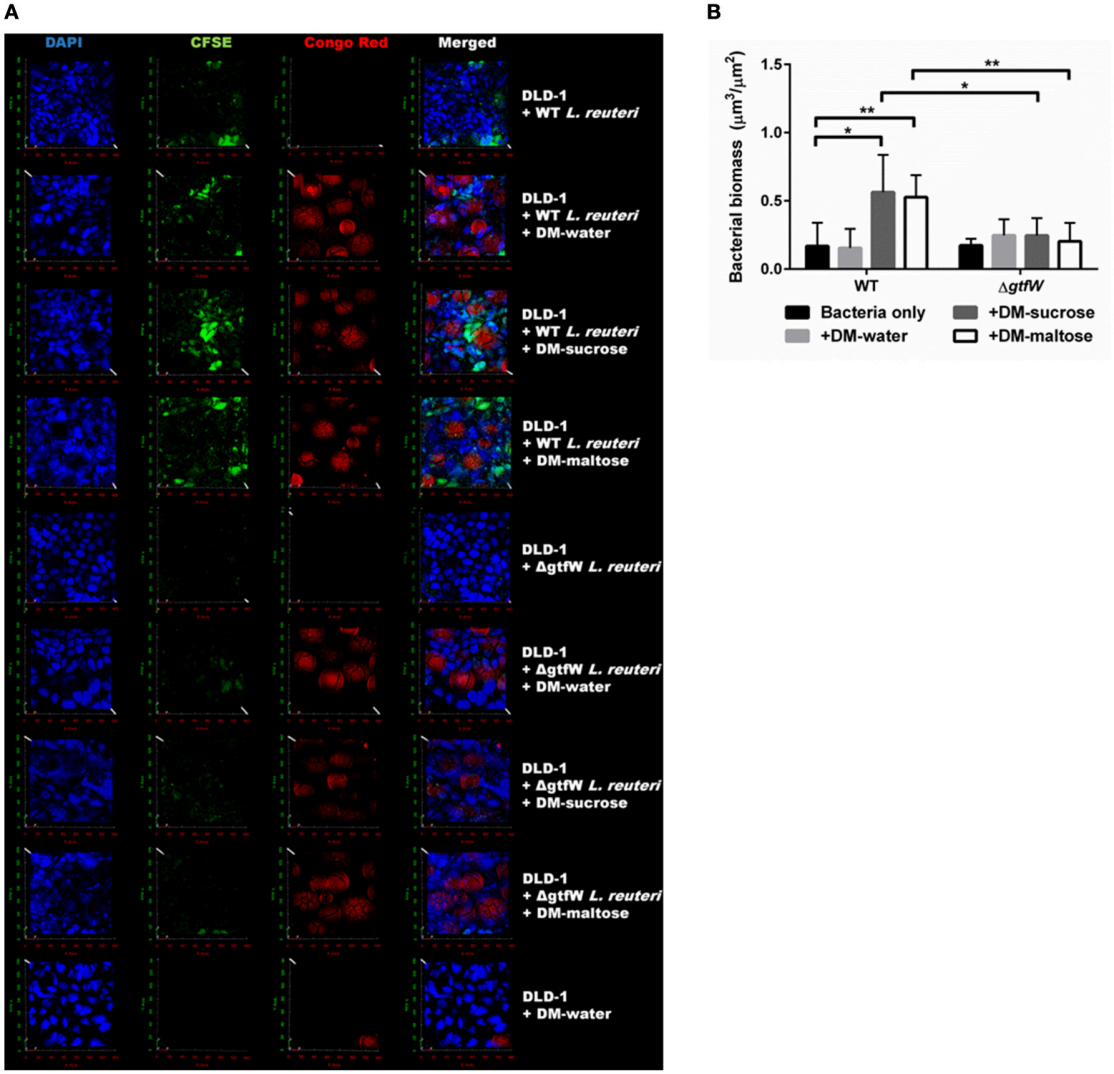
**Increased adherence to DLD-1 colonic epithelial cells is observed when ***L. reuteri*** was delivered as a biofilm attached to DMs. (A)** *In vitro* CLSM of DLD-1 epithelial cells (blue, DAPI), *L. reuteri* (green, CFSE), and DMs (red, Congo Red). WT *L. reuteri* (top four rows) compared to Δ*gtfW L. reuteri* (middle four rows) and no *L. reuteri* (bottom row). Bacteria and DMs were pre-stained, incubated for 1 h on pre-stained DLD-1 epithelial cells, washed three times, and fixed for CLSM analysis. **(B)** Comparison of bacterial biomass quantified via COMSTAT analysis of the green channel of CLSM images of WT and Δ*gtfW L. reuteri*. WT without DMs (*n* = 10) resulted in less total bacterial signal compared to either WT + DM-sucrose (*n* = 10) or WT + DM-maltose (*n* = 10). Δ*gtfW* showed no difference in relative number of bacteria adhered to DLD-1 cells, regardless of the presence of DMs. Error bars represent standard error of the mean. Statistical significance is indicated by the following: ^*^P < 0.05, ^**^P < 0.01.

To further show that DM luminal cargo of maltose and sucrose improved relative adherence of *L. reuteri* to epithelial cells *in vitro*, we analyzed WT and Δ*gtfW L. reuteri* adherence after 1 h incubation on DLD-1 cells visually, using CSLM (Figure 8). As with the CFU data presented in Figure 7, delivery of WT *L. reuteri* as a biofilm on maltose or sucrose-loaded DMs supported greater adherence to the DLD-1 cells than those delivered on water-loaded DMs or with no DMs, both by visual inspection (Figure 8A) and when analyzed by quantification of bacterial biomass using COMSTAT analysis of CSLM images (Figure 8B). The observed adherence was significantly diminished in the Δ*gtfW* mutant compared to the wild type, consistent with measured CFUs (Figure 7A).

Finally, we tested the effect of DM adhered WT *L. reuteri*'s ability to bind to mucin. While cellular binding of probiotics likely plays a role in colonization, a healthy GI tract has a mucus layer on the apical surface of epithelial cells, of which the primary constituent is mucin (Turner, 2009). Indeed it is believed that healthy commensals are found primarily within this layer so it is imperative that our formulation maintains it enhanced probiotic effects in the presence of mucin. As mucin adherence is not GTF-dependent, but rather controlled by specific mucin-binding proteins (Miyoshi et al., 2006; Lukic et al., 2012), we hypothesized that being bound to DMs would not have an effect on the ability of *L. reuteri* to adhere to mucin. As shown in Figure S8, there is no significant difference in relative adherence of WT *L. reuteri* to mucin when delivered as either a planktonic bacterial suspension or as a biofilm adhered to DMs after a 60 min incubation on mucin agar plates.

## Discussion

We have previously shown that a single dose of *L. reuteri* delivered as a biofilm adhered to DMs reduces the incidence of necrotizing enterocolitis (NEC) by 50% (Olson et al., 2016) in a rat pup model. Here we showed that *L. reuteri* bound to DMs with appropriate luminal cargo promoted significantly increased survival at low pH and supported increased adherence to human epithelial cells *in vitro*. Importantly *L. reuteri* and DMs are considered “generally recognized as safe” (GRAS) by the FDA. In fact, DMs have been used in medical products that are left in the body for long periods of time (years) with no ill effects (Hoy, 2012), such as with Debrisan®, a cicatrizant wound dressing (Jacobsson et al., 1976), Deflux®, a bulking gel used to treat vesicoureteral reflux (VUR) in children (Stenberg and Lackgren, 1995), and Solesta™, a bulking gel injected submucosaly into the anal canal to treat fecal incontinence (Hoy, 2012). The scope of the research presented here shows a small subset of possible beneficial cargos that can be placed into the DM lumen for utilization by *L. reuteri*, and for many applications it may be as simple as matching the correct lumen cargo precursor to the desired *L. reuteri*-produced effect (e.g., reuterin and histamine). Moreover, this formulation obviates recombinant versions of probiotics, an approach not currently approved by the FDA (Venugopalan et al., 2010).

An exciting feature of our novel formulation is the ability to directly deliver beneficial compounds to the probiotic bacteria that are adhered to the DM surface as a biofilm (Figure 1). To combine beneficial compounds (prebiotics) with beneficial bacteria to stimulate growth is a well-established concept in probiotic research and commercial applications (Collins and Gibson, 1999; de Vrese and Schrezenmeir, 2008). There is significant evidence to show that synergism between probiotics and prebiotics effectively increases the overall population of probiotic bacteria (de Vrese and Schrezenmeir, 2008; van Zanten et al., 2014) and promotes effective treatments of diseases such as inflammatory bowel disease (Geier et al., 2007) and necrotizing enterocolitis (Asmerom et al., 2015). However, a major drawback of traditional prebiotics is that they are typically limited to carbohydrates that are non-digestible or absorbable by the host to ensure sufficient availability to the probiotic bacteria in the gut. Our delivery system effectively solves this problem in that the probiotic bacterium *L. reuteri* is now delivered: (1) in association with DMs to which it adheres in greater numbers; (2) in the form of a biofilm which confers resistance to clearance; (3) along with a cargo of nutrients that promotes bacterial growth; (4) with cargos that promote production of the antimicrobial reuterin or histamine; (5) in a format that is resistant to acid-mediated killing thus promoting improved survival during transit through the acidic stomach, and (6) in a manner that appeared to better support adherence to intestinal epithelial cells and thus likely to promote persistence in the gut. With regard to *L. reuteri-*induced release of substance potentially beneficial to the host, reuterin has been suggested to inhibit competition by other gut flora, and histamine has been shown to have anti-inflammatory effects. Although the secondary metabolites produced from glycerol metabolism to generate reuterin (e.g., acrolein) and histamine could result in adverse effects at high levels, the maximum quantities generated with our formulations are <1% and <40% less than what is thought to be problematic in humans for acrolein (Figure S7) and histamine, respectively (Maintz and Novak, 2007; Thomas et al., 2012; Engels et al., 2016). Ongoing and future experiments utilizing *L. reuteri* adhered to DMs will test the putative aforementioned beneficial cargos in an *in vivo* animal model (Olson et al., in preparation) to demonstrate both safety and efficacy. Concurrently we are also investigating strategies for long-term storage and downstream application and delivery of our DM-based formulation.

Using maltose as cargo have particular value for several reasons; it is the substrate for this strain of *L. reuteri*'s glucosyltransferase (GTFW) (Leemhuis et al., 2013; Bai et al., 2015), induces *L. reuteri* to aggregate in a GTF-dependent manner (Walter et al., 2008), and causes *L. reuteri* to grow significantly faster and to a higher cell density (CFU/ml). In this study, we show that both maltose and sucrose have a positive effect on *L. reuteri* adherence to microspheres, promote adherence of *L. reuteri* to human intestinal epithelial cells, and improves bacterial survival in gastric acid (Figures 2, 3, 6, 7, 8). We have demonstrated in concurrent work that *S. mutans* binds rapidly and with high affinity to DMs, and the effect is increased in the presence of sucrose in a GTF-dependent manner (Mashburn-Warren et al., submitted). *S. mutans* and *L. reuteri* GTF proteins are very similar in sequence and structure. Sucrose is the sole substrate for *S. mutans* and most *L. reuteri* GTF proteins (Tieking et al., 2005; Walter et al., 2008), and sucrose has previously been shown to cause *L. reuteri* cultures to aggregate rapidly in a GTF-dependent manner (Walter et al., 2008). The positive effect of sucrose to induce GTFW dependent adhesion is likely due to GTFW acting as an adhesin to DMs (via the glucan binding domain) and sucrose's ability to induce *gtfW* expression (Figure S3A). Indeed, failure of sucrose to affect *L. reuteri* adherence to CMs (cross-linked glucan with variant glycosidic linkages) supports this notion. Sucrose-dependent biofilm formation has previously been linked to two-component regulatory systems in the rodent strain 100-23 of *L. reuteri* (Frese et al., 2011; Su and Ganzle, 2014); however, the genes necessary for this phenomenon appear to be absent in the human-derived strain of *L. reuteri* used in this study (23272/DSM 20016). Since sucrose is a preferred carbon source of the *L. reuteri* used in this study via its sucrose phophorylase mediated metabolism (Ganzle and Follador, 2012) it was not surprising that sucrose had a positive impact on biofilm formation and increased adherence to DMs and is likely due to the increased doubling time of *L. reuteri* in the presence of sucrose. The failure of glucose (a carbon source but not a *gtfW* inducer or GTFW substrate) and fructose [an inducer of *gtfW*, but not a carbon source (Figure S3 and data not shown), or substrate for GTFW] to enhance adherence to DMs suggests that understanding bacterial physiology will be critical in selecting beneficial luminal cargos.

Although we describe *L. reuteri* adhered to DMs as a biofilm, we have yet to characterize this physiologic state. We have demonstrated in previous work with the dental pathogen *Aggregatibacter actinomycetemcomitans*, that challenging a host with an already-established pathogenic biofilm results in greater ability of the pathogen to establish disease in an animal model of oral infection (Freire et al., 2011, 2017). While it is clear that being bound to DMs offers multiple advantages in terms of survivability and relative adherence to an epithelial target cell, the actual state of the DM-adhered *L. reuteri* and its phenotype has yet to be determined. There is evidence that microbes such as *L. reuteri* exist naturally as biofilms in the gastrointestinal tract (Macfarlane and Dillon, 2007), but there has thus far been a lack of research as to the composition and dynamics of biofilm communities of the gut (Hall-Stoodley et al., 2004; de Vos, 2015). *L. reuteri* adhered to DMs in our experiments are biofilms by definition and via our observations in Figure 6, which showed that bacteria adhered to DMs resisted low pH challenge better than planktonic bacteria. In experiments where *L. reuteri* bound to DMs are incubated with colonic cells we observed both aggregates of bacteria surrounding the DMs as well as those that were adhered to the confluent eukaryotic cell surface (Figure S9). Future work on the biofilm state will include the dlt operon that encodes proteins involved in D-alanylation of teichoic acid, and shown to be important in biofilm formation, adherence, and host colonization (Walter et al., 2007).

In this study we show that many parameters important to *L. reuteri*'s survivability and sustainability within the host can be improved by delivering *L. reuteri* as a biofilm on the surface of DMs that contain beneficial cargo. With more viable bacteria available after low pH challenge and supporting increased adherence to intestinal epithelial cells, the resulting expansion of probiotic bacteria available within the host should have an increased potentially beneficial effect. Further, we are able to deliver targeted nutrients and substrates directly to the bacteria adhered on the DM surface, which has broad-reaching implications for the type of compounds that can be co-delivered with orally consumed *L. reuteri*, which to date have been limited to carbohydrates that are indigestible by the host. Taken together, our novel delivery system provides an exciting framework for future probiotic development and deployment.

## Author contributions

SG, LM, and JN designed the study. JN and LM performed the experimental work. JN, LM, and SG analyzed the data. JN prepared the manuscript; and LM, SG, MB, and LB contributed to the final manuscript.

## Funding

This work was conducted utilizing discretionary funds.

### Conflict of interest statement

The authors declare that the research was conducted in the absence of any commercial or financial relationships that could be construed as a potential conflict of interest.
