# Supplementary material for: Enhanced Probiotic Potential of Lactobacillus reuteri When Delivered as a Biofilm on Dextranomer Microspheres That Contain Beneficial Cargo
Source: Front Microbiol. 2017 Mar 27;8:489. doi: 10.3389/fmicb.2017.00489 (PMC5366311; doi:10.3389/fmicb.2017.00489)
Supplement: Supplementary file 1 [file Table1.PDF]

**Table S1. Physical properties of Sephadex® dextranomer microspheres**

| Gel type | Dry bead size (μm) | | Wet bead size (μm) | | Permeability K* | Fractionation [Mr] globular proteins | Fractionation [Mr] dextrans | Exclusion limit (Da) | Swelling factor (ml/g) |
|---|---|---|---|---|---|---|---|---|---|
| | Low | High | Low | High | | | | | |
| G-10 | 40 | 120 | 55 | 165 | 19 | 700 | 700 | > 700 | 2-3 |
| G-15 | 40 | 120 | 60 | 180 | 18 | 1,500 | 1,500 | > 1,500 | 2.5-3.5 |
| **G-25 superfine** | **10** | **40** | **17** | **70** | **9** | **1,000-5,000** | **100-100** | **> 5,000** | **4-6** |
| G-25 fine | 20 | 80 | 35 | 140 | 30 | 1,000-5,000 | 100-100 | > 5,000 | 4-6 |
| G-25 medium | 50 | 150 | 85 | 260 | 80 | 1,000-5,000 | 100-100 | > 5,000 | 4-6 |
| G-25 coarse | > 100 | # | 87 | 510 | 290 | 1,000-5,000 | 100-5,000 | > 5,000 | 4-6 |
| G-50 superfine | 20 | 50 | 20 | 80 | 13.5 | 1,000-30,000 | 500-10,000 | > 30,000 | 9-11 |
| G-50 fine | 20 | 80 | 34 | 208 | 36 | 1,000-30,000 | 500-10,000 | > 30,000 | 9-11 |
| G-50 coarse | 100 | 300 | 200 | 610 | 400 | 1,000-30,000 | 500-10,000 | > 30,000 | 9-11 |
| G-75 superfine | 20 | 50 | 22 | 143 | # | 3,000-70,000 | 1,000-100,000 | > 70,000 | 12-15 |
| G-75 | 40 | 120 | 90 | 280 | # | 3,000-80,000 | 1,000-50,000 | > 70,000 | 12-15 |
| G-100 superfine | 10 | 40 | 25 | 100 | # | 4,000-100,000 | 1,000-100,000 | > 100,000 | 15-20 |
| G-100 | 40 | 120 | 100 | 310 | # | 4,000-150,000 | 1,000-100,000 | > 150,000 | 15-20 |

* Darcy's Law: $U = K (\Delta P) (L^{-1})$

**U** = linear flow rate in cm/h; **ΔP** = pressure drop over bed in cm H20; **L** = bed height in cm; **K** = specific permeability constant of particle size and water regain

# not provided by manufacturer
