# Supplementary material for: Enhanced Probiotic Potential of Lactobacillus reuteri When Delivered as a Biofilm on Dextranomer Microspheres That Contain Beneficial Cargo
Source: Front Microbiol. 2017 Mar 27;8:489. doi: 10.3389/fmicb.2017.00489 (PMC5366311; doi:10.3389/fmicb.2017.00489)
Supplement: Supplementary file 2 [file Image1.PDF]

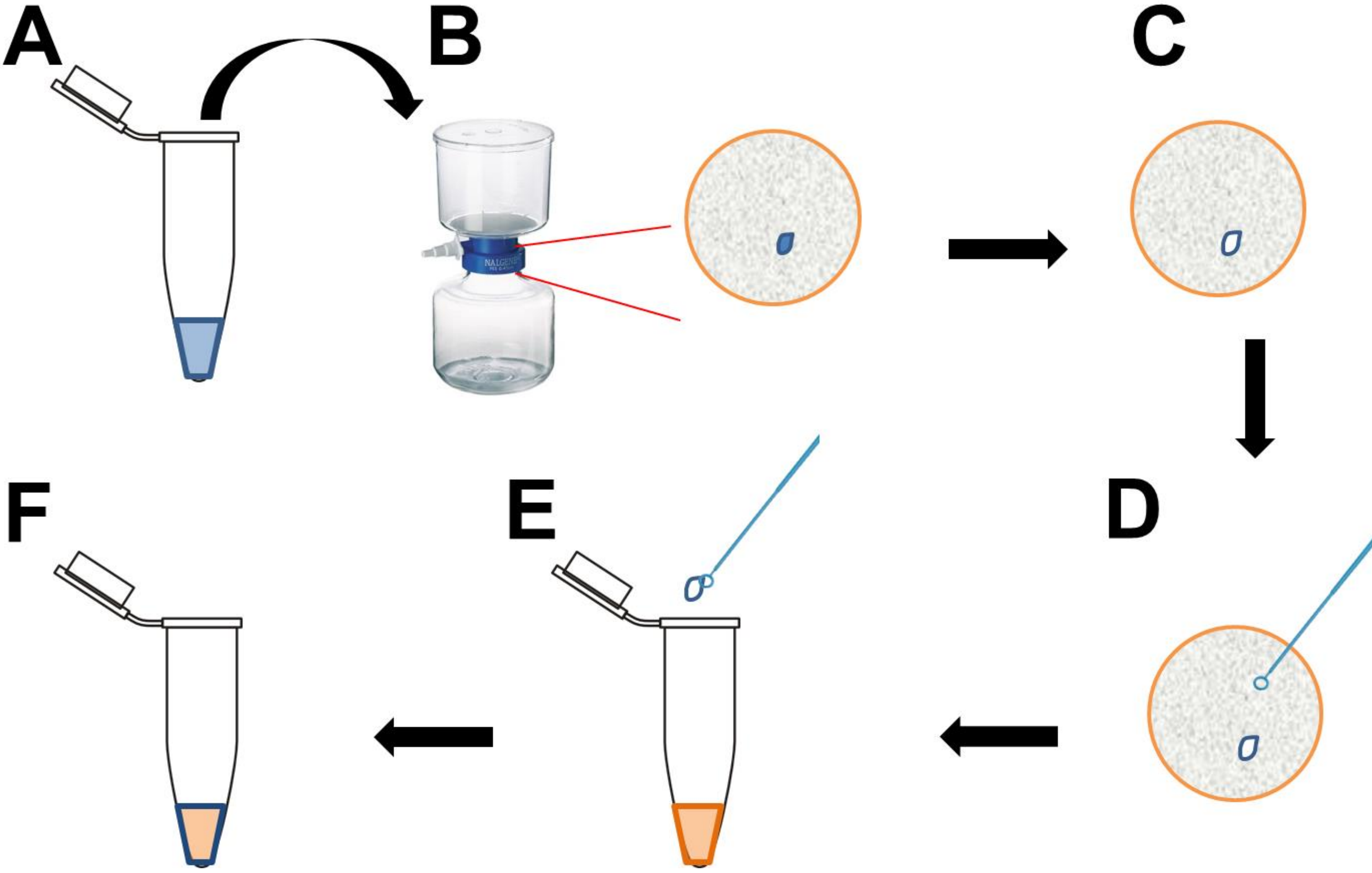

Figure S1. Illustration of DM cargo loading, filtration, and addition to bacterial culture. (A) Dehydrated DMs and desired cargo (e.g. 1M maltose) were incubated together to allow diffusion of solution into DMs. (B) The DM + solution is vortexed and pipetted to a vacuum filtration system. (C) The vacuum removes excess solution, leaving just DMs with absorbed cargo. (D) The DM-cargo pellet can now be removed from the vacuum filter by scraping with a sterile loop. (E) The DM-cargo pellet is transferred to a bacterial solution, typically bacteria resuspended in saline. (F) The final product is bacteria + DM-cargo together in solution, which can then be used for downstream applications (e.g. assays, oral gavage, etc.).
