# Supplementary material for: Enhanced Probiotic Potential of Lactobacillus reuteri When Delivered as a Biofilm on Dextranomer Microspheres That Contain Beneficial Cargo
Source: Front Microbiol. 2017 Mar 27;8:489. doi: 10.3389/fmicb.2017.00489 (PMC5366311; doi:10.3389/fmicb.2017.00489)
Supplement: Supplementary file 3 [file Image2.PDF]

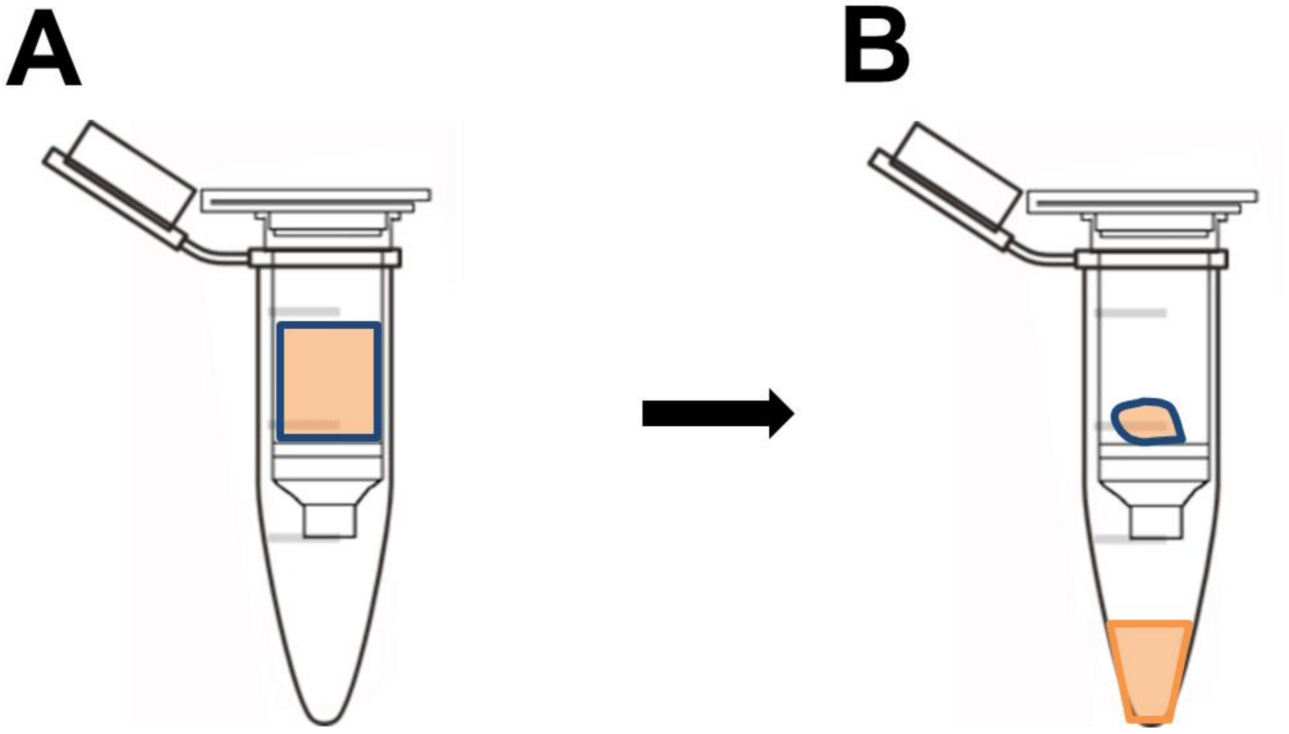

Figure S2. Illustration of spin column DM adherence assay. (A) A bacteria + DM-cargo mixture is incubated together on top of a spin column filter within a 1.5 or 2.0ml microcentrifuge tube. After the desired incubation time (e.g. 5 minutes), the tube + column is centrifuged at $\leq$100 x g to separate adhered and non-adhered bacteria to DMs. (B) After centrifugation, non-adhered cells will be in the flow through at the bottom of the microcentrifuge tube, and adhered bacteria to DMs will remain on the surface of the filter with the DMs (filter pore size is too small for DM passage, but small enough for bacterial cells). The cells present in the flow through are enumerated by serial dilution plating. A bacteria only (no DMs) control is used as a baseline, and all DM experiments are subtracted from the baseline.
