# Supplementary material for: Enhanced Probiotic Potential of Lactobacillus reuteri When Delivered as a Biofilm on Dextranomer Microspheres That Contain Beneficial Cargo
Source: Front Microbiol. 2017 Mar 27;8:489. doi: 10.3389/fmicb.2017.00489 (PMC5366311; doi:10.3389/fmicb.2017.00489)
Supplement: Supplementary file 4 [file Image3.PDF]

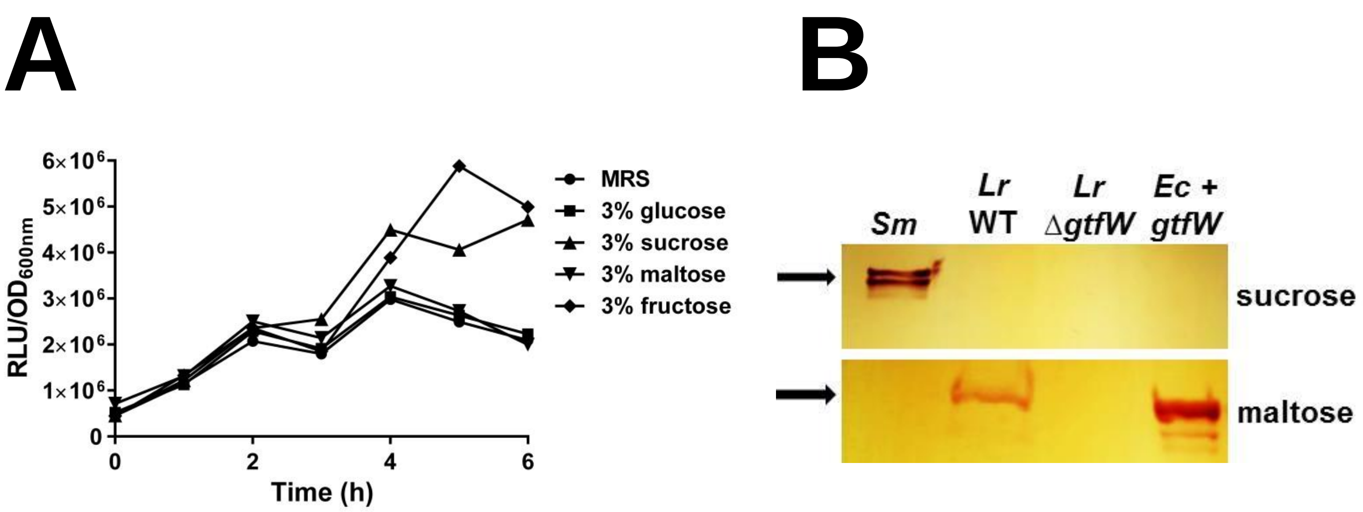

Figure S3. Sucrose induces *gtfW*, but maltose is the substrate for GTFW. (A) A *gtfW* transcriptional reporter was constructed by fusing the click beetle luciferase downstream of the *gtfW* promoter on a plasmid, followed by introduction into *L. reuteri* (strain LMW 501). Expression of *gtfW* was monitored throughout growth in MRS, with or without the indicated additions by removing a 100 µl aliquot every hour, and measuring the OD_{600nm}. An additional 80 µl aliquot was removed and added to 20 µl of 2 mM D-luciferin and allowed to incubate at RT for 5 min, followed by luminescence detection. (B) GTFW enzymatic activity. Proteins extracted from *S. mutans*, *L. reuteri* WT, *L. reuteri* Δ*gtfW* (strain LMW 500), and *E. coli* harboring *gtfW* on an inducible plasmid (*Ec*) (strain LMW 502), were subjected to SDS-PAGE followed by PAS staining to examine GTFW enzymatic activity. 5% sucrose or 5% maltose were used as substrates. The arrows indicate GTFW activity.
