# Supplementary material for: Enhanced Probiotic Potential of Lactobacillus reuteri When Delivered as a Biofilm on Dextranomer Microspheres That Contain Beneficial Cargo
Source: Front Microbiol. 2017 Mar 27;8:489. doi: 10.3389/fmicb.2017.00489 (PMC5366311; doi:10.3389/fmicb.2017.00489)
Supplement: Supplementary file 5 [file Image4.PDF]

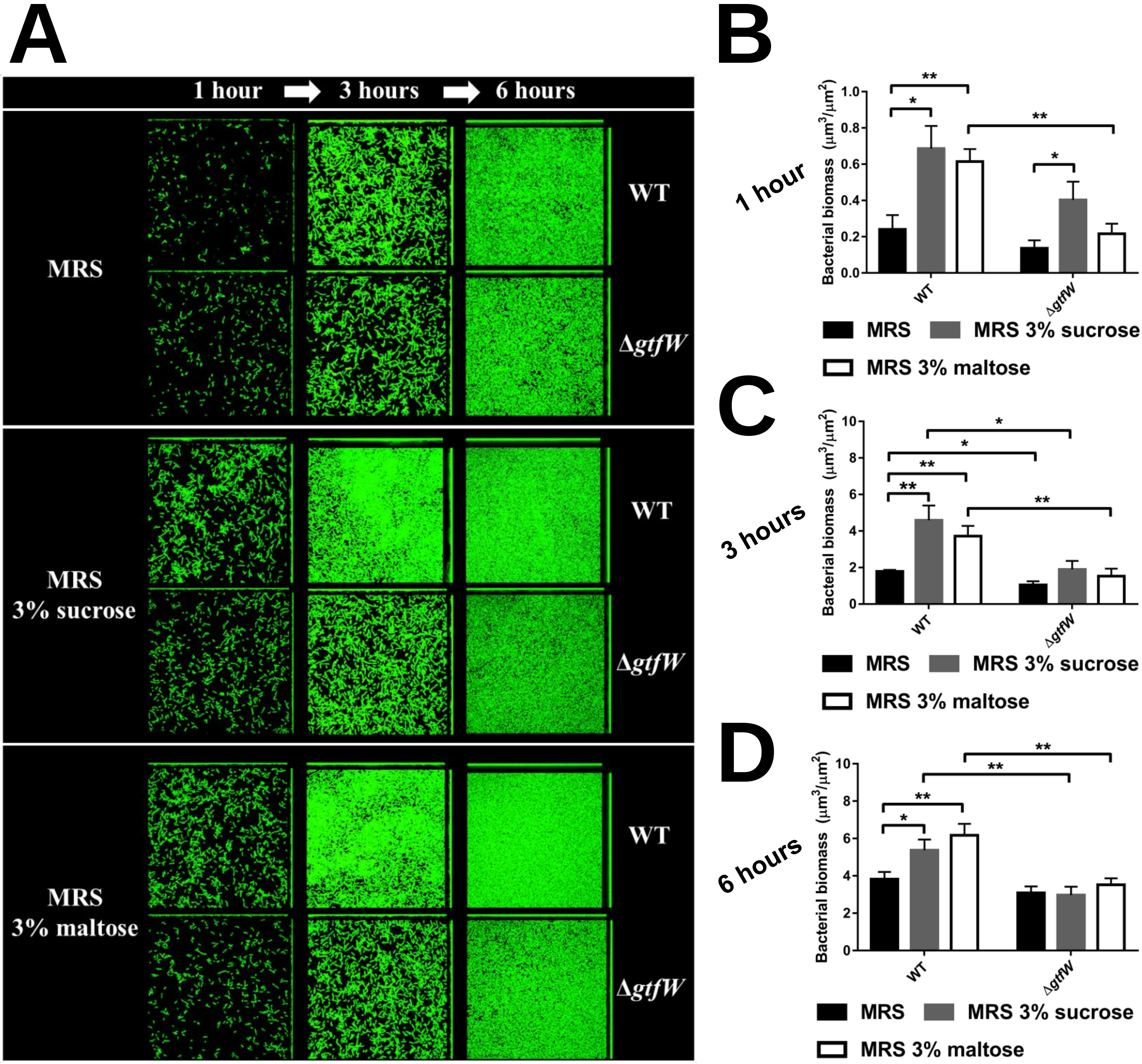

Figure S4. GTFW contributed to early biofilm formation in growth medium supplemented with sucrose or maltose. *L. reuteri* WT and Δ*gtfW* were seeded into 8-well borosilicate chamber slides and incubated for 1, 3, or 6 hours at 37°C 5% CO_2. At the designated time intervals, the bacteria were stained for viability with LIVE/DEAD stain, fixed, visualized via confocal microscopy (CLSM), and quantified via COMSTAT analysis of the fluorescent signal. (A) CLSM of *L. reuteri* biofilms at 1, 3, and 6 hours showed significantly more bacteria present and increased aggregation of WT bacteria in conditions with either sucrose or maltose at 1 hour compared to the *gtfW* mutant (left column), which was confirmed by quantification of the green fluorescent signal (B). The GTF-dependent increase in biofilm with sucrose or maltose present was increased after 3 hours (A - middle column, & C) and further increased after 6 hours (A – right column, & D). The *gtfW* mutant, being unable to utilize either maltose for biofilm formation, still benefited from sucrose in the growth medium after 1 hour, likely due to increased growth rate (data not shown). Error bars represent standard error of the mean. Statistical significance is indicated by the following: * $P < 0.05$, ** $P < 0.01$.
