# Supplementary material for: Enhanced Probiotic Potential of Lactobacillus reuteri When Delivered as a Biofilm on Dextranomer Microspheres That Contain Beneficial Cargo
Source: Front Microbiol. 2017 Mar 27;8:489. doi: 10.3389/fmicb.2017.00489 (PMC5366311; doi:10.3389/fmicb.2017.00489)
Supplement: Supplementary file 6 [file Image5.PDF]

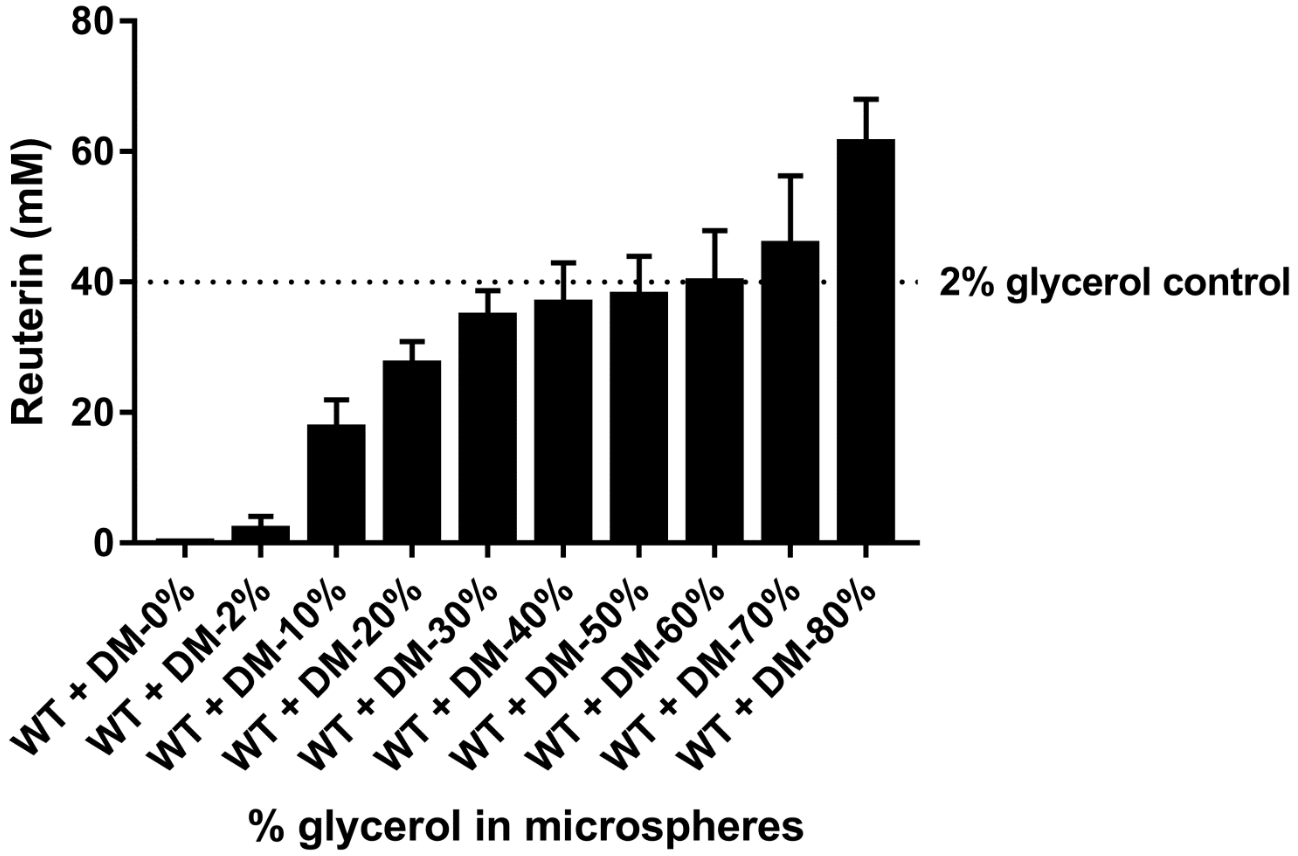

Figure S5. *L. reuteri* can produce reuterin from glycerol-loaded microspheres. *L. reuteri* incubated for 1 hour with DMs that contained 0-80% glycerol as the only source of glycerol in the experimental conditions were measured for relative reuterin production. For comparison, the amount of reuterin produced by *L. reuteri* without DMs in a 2% glycerol solution was used as a control (dotted line). Error bars represent standard error of the mean.
