# Supplementary material for: Enhanced Probiotic Potential of Lactobacillus reuteri When Delivered as a Biofilm on Dextranomer Microspheres That Contain Beneficial Cargo
Source: Front Microbiol. 2017 Mar 27;8:489. doi: 10.3389/fmicb.2017.00489 (PMC5366311; doi:10.3389/fmicb.2017.00489)
Supplement: Supplementary file 7 [file Image6.PDF]

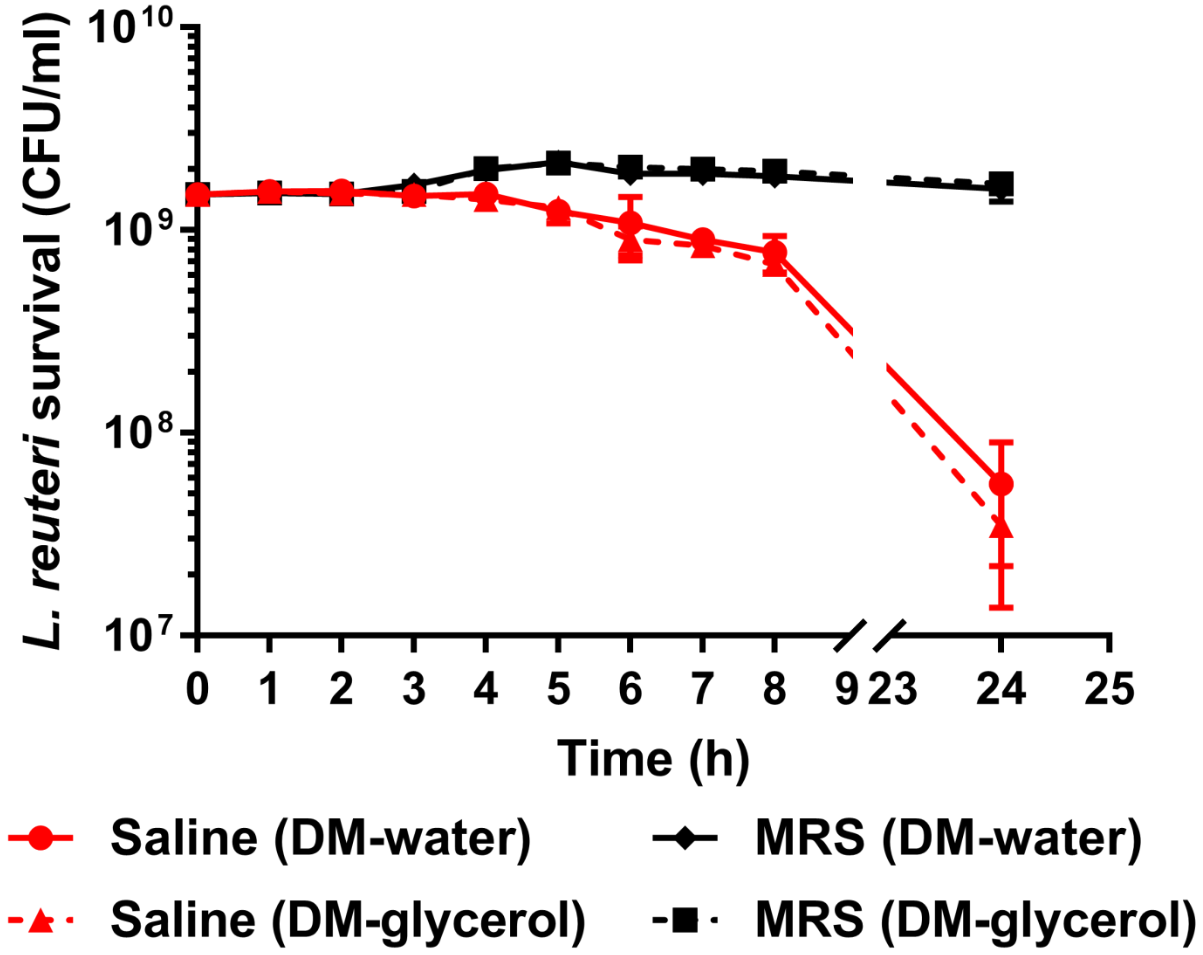

Figure S6. Glycerol delivered via DMs and any subsequently produced metabolites did not affect *L. reuteri* survival. Overnight cultures of WT *L. reuteri* were washed and resuspended in either saline or MRS medium. 5mg of DM-water or DM-80% glycerol were then added to *L. reuteri* and incubated at 37°C. At hourly intervals the aliquots were taken for subsequent serial dilution and plating for viable CFU. After 24 hours there was no significant difference between cultures incubated in the same medium (saline or MRS) with either DM-water or DM-80% glycerol.
