# Supplementary material for: Enhanced Probiotic Potential of Lactobacillus reuteri When Delivered as a Biofilm on Dextranomer Microspheres That Contain Beneficial Cargo
Source: Front Microbiol. 2017 Mar 27;8:489. doi: 10.3389/fmicb.2017.00489 (PMC5366311; doi:10.3389/fmicb.2017.00489)
Supplement: Supplementary file 8 [file Image7.PDF]

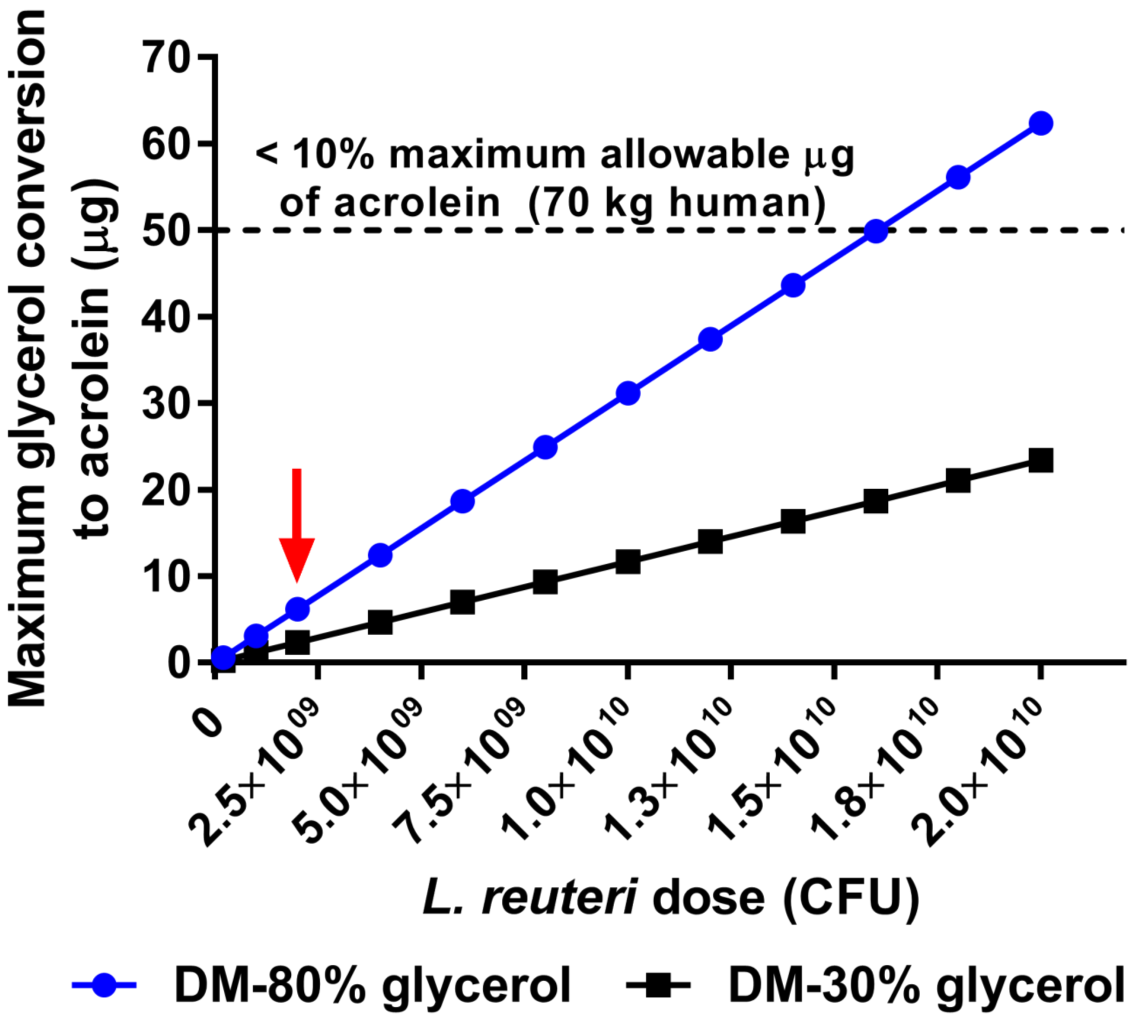

Figure S7. Maximum conversion of DM-provided glycerol to acrolein did not result in toxic levels of acrolein. The World Health Organization (WHO) recommends ingestion of no more than 7.5 µg/kg of body weight of acrolein per day. Assuming 100% conversion of available glycerol provided via DMs into acrolein by *L. reuteri*, the dosage of *L. reuteri* and DM-glycerol utilized in this work (red arrow) resulted in a maximum of 6.24 µg acrolein produced. The dashed line (50 µg acrolein) represents < 10% of the daily allowable amount of acrolein for a 70 kg human.
