# Supplementary material for: Enhanced Probiotic Potential of Lactobacillus reuteri When Delivered as a Biofilm on Dextranomer Microspheres That Contain Beneficial Cargo
Source: Front Microbiol. 2017 Mar 27;8:489. doi: 10.3389/fmicb.2017.00489 (PMC5366311; doi:10.3389/fmicb.2017.00489)
Supplement: Supplementary file 9 [file Image8.PDF]

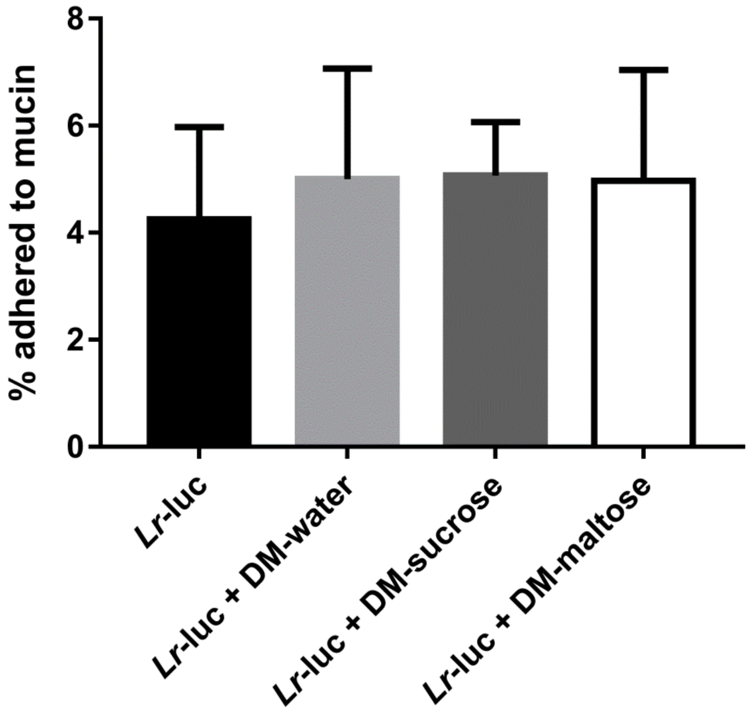

Figure S8. *L. reuteri* delivered as a biofilm on DMs does not inhibit adherence to mucin. *L. reuteri* reporter that expressed click beetle luciferase was dispensed either planktonically or as a biofilm on the DM surface onto agar plates that contained either 2% mucin + 0.8% agar or 0.8% agar, incubated at room temperature for 1 hour, then washed to remove non-adhered *L. reuteri*. D-luciferin (0.4 mM) was then added to the plates, and the plates were imaged for luminescent signal that originated from remaining adhered bacteria. To calculate the amount of bacteria adhered to only mucin, the relative luminosity of the agar-only plates was subtracted from the relative luminosity of the mucin + agar plates.
