# Supplementary material for: Enhanced Probiotic Potential of Lactobacillus reuteri When Delivered as a Biofilm on Dextranomer Microspheres That Contain Beneficial Cargo
Source: Front Microbiol. 2017 Mar 27;8:489. doi: 10.3389/fmicb.2017.00489 (PMC5366311; doi:10.3389/fmicb.2017.00489)
Supplement: Supplementary file 10 [file Image9.PDF]

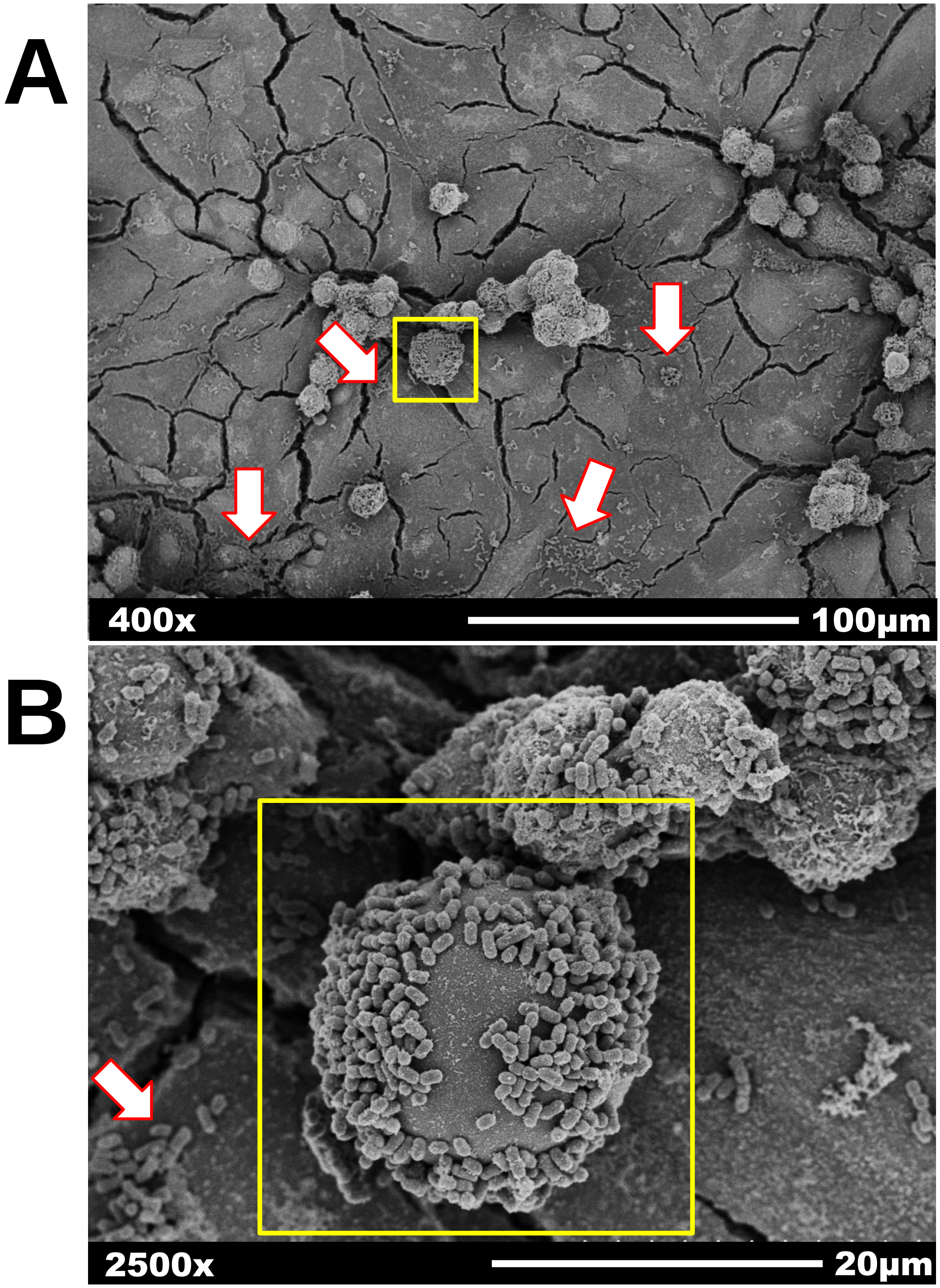

Figure S9. *L. reuteri* adhered to DMs and *L. reuteri* attached to the surface of DLD-1 human colonic epithelial cells. *In vitro* SEM of *L. reuteri* and DMs on a confluent monolayer of DLD-1 cells. Bacteria and DMs were incubated for 1 hour on DLD-1 epithelial cells, washed three times, fixed and prepared for SEM analysis. 400X (A) and 2500X (B) magnification showed *L. reuteri* adhered to a DM (yellow box) and several clusters of *L. reuteri* without DMs (white arrows) adhered to the surface of DLD-1.
